# Molecular epidemiology of the citrus bacterial pathogen *Xanthomonas citri* pv. *citri* from the Arabian Peninsula reveals a complex structure of specialist and generalist strains

**DOI:** 10.1111/eva.13451

**Published:** 2022-08-26

**Authors:** Olivier Pruvost, Yasser Eid Ibrahim, Anwar Hamoud Sharafaddin, Karine Boyer, Arya Widyawan, Mohammed Ali Al‐Saleh

**Affiliations:** ^1^ CIRAD, UMR PVBMT Saint Pierre France; ^2^ Department of Plant Protection, College of Food and Agriculture Sciences King Saud University Riyadh Saudi Arabia

**Keywords:** bacterial disease, citrus, host specialization, microsatellites, minisatellites, molecular epidemiology

## Abstract

Molecular epidemiology studies are essential to refine our understanding of migrations of phytopathogenic bacteria, the major determining factor in their emergence, and to understand the factors that shape their population structure. Microsatellite and minisatellite typing are useful techniques for deciphering the population structure of *Xanthomonas citri* pv. *citri*, the causal agent of Asiatic citrus canker. This paper presents a molecular epidemiology study, which has improved our understanding of the history of the pathogen's introductions into the Arabian Peninsula, since it was first reported in the 1980s. An unexpectedly high genetic diversity of the pathogen was revealed. The four distinct genetic lineages within *X. citri* pv. *citri*, which have been reported throughout the world, were identified in the Arabian Peninsula, most likely as the result of multiple introductions. No copper‐resistant *X. citri* pv. *citri* strains were identified. The pathogen's population structure on Mexican lime (their shared host species) was closely examined in two countries, Saudi Arabia and Yemen. We highlighted the marked prevalence of specialist pathotype A* strains in both countries, which suggests that specialist strains of *X. citri* pv. *citri* may perform better than generalist strains when they occur concomitantly in this environment. Subclade 4.2 was the prevailing lineage identified. Several analyses (genetic structure deciphered by discriminant analysis of principal components, R_ST_‐based genetic differentiation, geographic structure) congruently suggested the role of human activities in the pathogen's spread. We discuss the implications of these results on the management of Asiatic citrus canker in the region.

## 1 INTRODUCTION

Agricultural industries are threatened by plant disease emergences, a consequence of many factors, including the globalization of plant product trade, a marked increase of human international travel, crop intensification linked to the increase in human population size and climate change (Anderson et al., 2004; Paini et al., 2016; Savary et al., 2019). Refining our understanding of the emergence, spread, and evolution of plant pathogenic populations requires a thorough comprehension of the determinants of their fitness and their eco‐evolutionary dynamics at the within‐host up to agricultural landscape scales in response to environmental selective pressures (McDonald & Stukenbrock, 2016; Penczykowski et al., 2016; Plantegenest et al., 2007). Bacterial pathogens exhibit a highly variable degree of host specialization and can be broadly divided into specialists versus generalists on the basis of their host range width (Baumler & Fang, 2013; Shaw et al., 2020). The degree of host specialization of pathogens has major implications for disease progress and management. Heterogeneous agricultural landscapes have a negative impact on the natural dispersal of highly specialized pathogens (McDonald & Stukenbrock, 2016; Plantegenest et al., 2007). Moreover, the degree of host specialization of a pathogen markedly influences surveillance strategies (Gandon et al., 2013; Morris et al., 2022; Parnell et al., 2017). In the specific case of some perennial crops affected by highly specialized pathogens, scion replacement using top grafting strategies can be efficient management options (Mudge et al., 2009). Among factors involved in the emergence of pathogens, the impact of host specialization on disease dynamics and epidemiology is a major issue. Population biology, which has been very successful in furthering our knowledge on the dynamics and evolution of single‐host pathogens, has the ability to refine the understanding on the key question of the ecological and evolutionary advantages and disadvantages of host specialization (Woolhouse et al., 2001). There is a strong need for more experimental data to (i) evaluate the performance of specialist vs. generalist pathogens in situations where they occur concomitantly and (ii) compare their fitness (Barrett et al., 2009).

Citrus is an important crop worldwide (FAS, 2019). For several decades, it has been threatened by various diseases. One of the most severe, Citrus bacterial canker (CBC), has damaged the citrus industries and is now endemic in many hot‐humid citrus growing regions of the world (Graham et al., 2004). CBC has a wide host range among citrus species and relatives (Gottwald et al., 1993). Canker symptoms occur on all aerial organs and severe CBC causes fruit blemishing, defoliation, and early fruit drop (Gottwald et al., 2002). Two pathovars of *Xanthomonas citri* (i.e., infra‐specific groups of bacteria with similar pathological characteristics such as host range and disease syndrome) cause visually indistinguishable canker‐like symptoms on some *Citrus* species and sometimes on other rutaceous genera.

*Xanthomonas citri* pv. *citri*, the causal agent of Asiatic citrus canker (ACC), infects a single plant family, Rutaceae. In agricultural terms, this is by far the most significant form of CBC (Graham et al., 2004). CBC is an agriculturally major citrus disease worldwide. It causes direct (premature leaf and fruit drop, decrease in fruit quality and yield) and indirect losses (restricted access to export markets, increase in production costs due to implemented surveillance and management options, undesirable side effects of pesticide application), thereby resulting in a significant socioeconomic impact on impacted citrus industries (Graham et al., 2004; Stall & Seymour, 1983). The annual cost of CBC in Florida only (approximately 0.3 million hectares of commercial citrus at the time of assessment) was estimated as ca. 350 million US$ (Gottwald et al., 2002). Strains of *X. citri* pv. *citri* differ in host range. Three pathotypes (A, A* and A^w^), i.e., groups of strains causing canker on a distinct range of citrus species and/or distinct defense phenotypes on non‐host citrus, were described (Rybak et al., 2009; Sun et al., 2004; Vernière et al., 1998). Pathotype A can cause canker on all *Citrus* lines and several species of other rutaceous genera. It was reported in most countries where ACC was recorded. In contrast, pathotypes A* and A^w^ have a narrow host range primarily restricted to Mexican lime (*C*. x *aurantiifolia*) under natural conditions. Outbreaks caused by pathotype A* strains have been reported in Asia, the Arabian Peninsula, and East Africa. Some A* strains reported in Iran produce mild canker when inoculated into other citrus species (Derso et al., 2009; Escalon et al., 2013; Vernière et al., 1998). Pathotype A^w^ has the unique feature of inducing a hypersensitive reaction (HR) when inoculated at high titer into some non‐host citrus lines, such as grapefruit (*C* x *paradisi*) and sweet orange (*C*. x *sinensis*) (Rybak et al., 2009; Sun et al., 2004). This phenotype was found to be associated with the specific presence in A^w^ strains of the type III effector *avrGf1* (Rybak et al., 2009; Webster et al., 2020). This suggests that the genetic basis of the restricted host range in A* and A^w^ is likely to be different. Although pathotype A^w^ strains were first reported in Florida, it is now thought that they originated in India (Bui Thi Ngoc, Vernière, Jarne, et al., 2009; Schubert et al., 2001). While A* and A^w^ strains have a globally lower economic impact due to their narrow host range, they are still economically important in countries with a well‐developed lime industry. For example, extensive cankers and dieback caused by A* strains were observed on Mexican lime in Thailand (Bui Thi Ngoc et al., 2007).

Another pathogen, *X. citri* pv*. aurantifolii*, causing a similar disease referred to as South American canker, has a much more limited impact on citrus industries (Rossetti, 1977). *X. citri* pv. *aurantifolii* and *X. citri* pv. *citri* can be readily distinguished with several molecular detection or genotyping assays (Almeida et al., 2010; Bui Thi Ngoc et al., 2010; Cubero & Graham, 2002; Mavrodieva et al., 2004; Robène et al., 2020).

Given the high agricultural significance of *X. citri* pv. *citri*, numerous genotyping techniques for subtyping outbreak strains have been developed over the years (Bui Thi Ngoc, Vernière, Jarne, et al., 2009, Bui Thi Ngoc, Vernière, Vital, et al., 2009; Cubero & Graham, 2002; Jeong et al., 2019; Pruvost et al., 2014). Only some of the techniques are powerful. Minisatellite and CRISPR genotyping are currently the most suitable techniques for locating outbreak strains in relation to the global diversity of *X. citri* pv. *citri* (Jeong et al., 2019; Pruvost et al., 2014). Online databases make it possible to compare new outbreak strains with reference strains (http://www.biopred.net/mlva/). These techniques may be valuable alternatives to whole genome sequencing (WGS) for surveillance of not highly critical cases. They can also be helpful in countries where bioinformatic analysis of WGS data is not straightforward. Microsatellite genotyping has also proved useful in molecular epidemiology studies in order to (i) assess the population structure of *X. citri* pv. *citri* at micro geographical scales, (ii) characterize new subclades, (iii) identify pathways associated with pathogen spread, and (iv) decipher invasion routes (Leduc et al., 2015; Pruvost et al., 2015, 2019; Vernière et al., 2014). To date, all these studies have analyzed outbreaks associated with a single pathotype. The molecular methods outlined above have the potential to analyze more complex epidemiological situations, where several pathotypes infect Mexican lime and/or its very close relative, alemow (*C*. x *macrophylla*).

Citrus bacterial canker became successfully established in several countries in the Arabian Peninsula in the 1980s. In this region, several *Citrus* species are commercially produced. The citrus industries predominantly grow Mexican lime and sweet orange, which are widely established on the peninsula. CBC was first observed in 1982 in the Yemen Arab Republic (North Yemen; Dimitman, 1984). The disease status in Yemen at the time was based solely on visual observations of Mexican lime and sour orange (*C. x aurantium*), two host species of *X. citri* pv*. aurantifolii* and *X. citri* pv. *citri* (Rossetti, 1977). It was hypothesized that the putative pathogen was introduced into Yemen via contaminated citrus trees imported from India in 1981 (Dimitman, 1984). CBC was first observed in Saudi Arabia on Mexican lime at the end of 1983, in Sabya (Jizan region). It was thought to have originated from Yemen (Fadlallah, 1986). Additional observations of the disease were recorded from 1985 on the same species in several citrus nurseries of the Najran region and three years later in orchards (Fadlallah, 1986). The pathogen isolated from these regions at that time was identified as *X. citri* pv. *citri* pathotype A* (Vernière et al., 1998). A more recent study suggested that both pathotypes A and A* occur in Saudi Arabia (Al Saleh et al., 2014). Using a detached leaf assay, a recent study confirmed that there was some variation in pathogenicity on grapefruit among strains from Saudi Arabia (Ibrahim et al., 2019). As both pathotype A and a subclade of pathotype A* were found to produce lesions on grapefruit after inoculation (Escalon et al., 2013), Ibrahim et al. (2019) concluded that precise genotyping data is needed to determine whether or not pathotype A occurs in Saudi Arabia. CBC was reported in 1984 in the United Arab Emirates in the Dhaid area (Bové, 1995). Strains obtained from this outbreak produced a virulence pattern similar to that of pathotype A strains (El Goorani, 1989). Lastly, CBC was first observed in Oman in 1986 on Mexican lime and grapefruit in two remote areas located near Ibri and Salalah, respectively (Bové, 1995). Bacterial strains isolated at that time were highly diverse and were assigned to all pathotypes reported to date (A, A*, and A^w^). Besides India, Oman is the sole country that hosts all three pathotypes of *X. citri* pv. *citri* (Gordon et al., 2015). Attempts to eradicate the pathogens were recommended in the four countries at that time (Bové, 1995). However, the action plans were ineffective and the pathogen has continued to spread geographically at least in Saudi Arabia (Ibrahim et al., 2017).

In the context of (i) the high regional genetic diversity of *X. citri* pv. *citri* (Gordon et al., 2015; Pruvost et al., 2015) and (ii) uncertainties with regard to the lineage(s) behind the emergence of CBC on the Arabian Peninsula, the present study set out to characterize the CBC‐causing pathogen(s) in Saudi Arabia and Yemen, with special emphasis on Mexican lime (i.e., a widely distributed citrus species that can host all *X. citri* lineages reported to date and on which CBC is highly prevalent). We conducted a precise genetic and pathological assessment of the diversity of Saudi and Yemeni strains. Drawing on a recent comprehensive strain collection, supplemented with strains collected during the first outbreaks in the 1980s in Saudi Arabia, Yemen, and Oman, we compared them to an international collection of reference strains. Here, we used genotyping data to refine the understanding of the histories of CBC establishments in Saudi Arabia and Yemen and confirmed the presence of pathotypes A and A* in both countries (Dimitman, 1984; Ibrahim et al., 2019). This complex epidemiological situation prompted an extensive sampling on Mexican lime on which we deciphered the distribution and genetic structure of several populations differing in host range, and highlighted a marked prevalence of specialist strains in this environment.

## 2 MATERIALS AND METHODS

### 2.1 Surveyed areas

**FIGURE 1:**
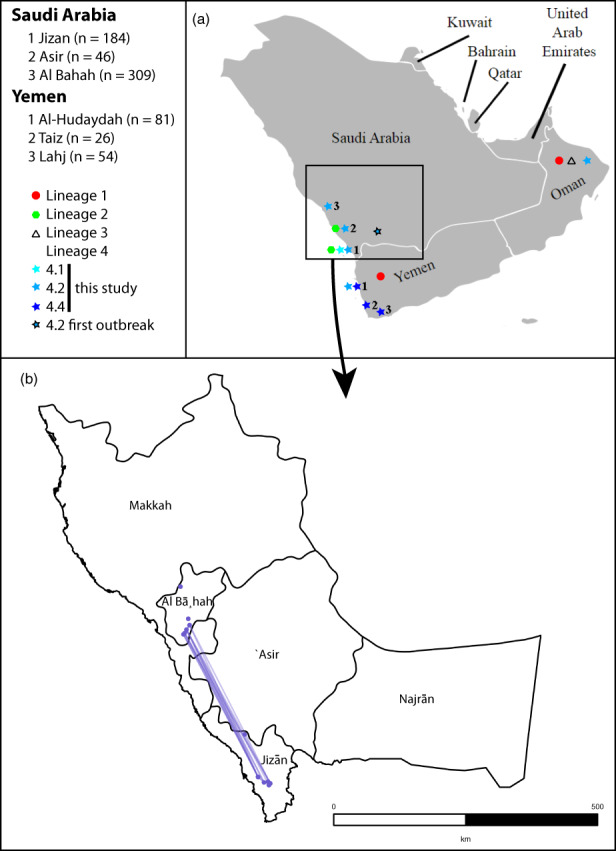
(a) Map of the Arabian Peninsula showing the three regions from Saudi Arabia and the three governorates from Yemen where citrus canker samples were collected (including the number of authenticated *Xanthomonas citri* pv. *citri* strains and their genetic assignation). Note that the exact isolation place is unknown for Omanese strains from a previous study (Vernière et al., 1998). (b) Close‐up map. Blue solid lines link pairs of subclade 4.2 local populations (blue dots) for which no significant genetic differentiation (*p* > 0.05 based on R_ST_) was found.

Surveys on CBC were conducted in Saudi Arabia (SA) and Yemen (YE) in citrus‐growing areas. We surveyed: thirty‐two citrus blocks on commercial farms and four citrus nurseries in Al‐Baha, Al‐Madina, Aseer, and Jizan in Saudi Arabia; and twenty‐one blocks on commercial farms in Al‐Hudaydah, Lahj, and Taiz in Yemen (Figure 1a and Table S1). When available, ten citrus leaves with putative CBC symptoms were randomly collected from each tree at each site (30 trees/site). Samples were sealed in plastic bags and taken to the laboratory at the King Saud University (College of Food and Agriculture Sciences, Plant Protection Department) for the culture of *Xanthomonas* spp.

### 2.2 Isolation of bacterial strains

Infected leaves were washed in running water and surface‐sterilized by wiping with 75% ethanol. Bacteria were isolated from diseased leaf tissues. This involved cutting leaves with canker lesions into small pieces, crushing them in sterile distilled H_2_O and leaving them for 10 min at room temperature. One loopful of the bacterial suspension was streaked onto KC semi‐selective medium containing yeast extract 7 g L^−1^, peptone 7 g L^−1^, glucose 7 g L^−1^, agar 18 g L^−1^, cephalexin 40 mg L^−1^, kasugamycin 20 mg L^−1^, propiconazole 20 mg L^−1^; pH 7.2 (Pruvost et al., 2005). The plates were incubated at 28°C for 3 days. Single bacterial colonies displaying characteristics of *Xanthomonas* spp. were selected and purified by re‐streaking on YPGA plates (yeast extract 7 g L^−1^, peptone 7 g L^−1^, glucose 7 g L^−1^, agar 18 g L^−1^; pH 7.2) for 24 h at 28°C. The bacterial strains were stored in 50% glycerol at −80°C until use. Although strains originated from several *Citrus* species, Mexican lime was sampled more extensively (Table S1). Whenever possible, local populations (i.e., collection including ≥14 strains originating from distinct plants sampled from a single grove or nursery; *n* = 23) were assembled. Twenty‐four and three strains isolated in the 1980s from Saudi Arabia and Yemen, respectively, were included as reference.

### 2.3 Phenotypic characterization

Pure cultures of putative *Xanthomonas* spp. were assayed using biochemical and physiological tests, including gelatine and casein hydrolysis and growth in the presence of 3% NaCl, as described by Vernière et al. (1991). A subset of 36 strains (Table S1) were assayed for (i) copper resistance, as described previously (Richard et al., 2017) and pathogenicity on Mexican lime and mandarin cv. Kinnow, using the attached leaf assay. Briefly, adult leaves from the youngest vegetative flush were infiltrated with bacterial suspensions containing ca. 1.10^5^ cfu mL^−1^. Disease development was scored weekly. Technical details are available in previous studies (Escalon et al., 2013; Vernière et al., 1998).

### 2.4 Polymerase chain reaction (PCR) assay

The bacterial strains used in this study are listed in Table S1. Bacterial cultures were grown in YP broth tubes (yeast extract 7 g L^−1^; peptone 7 g L^−1^; pH 7.2) and incubated for 16–18 h at 28°C in an orbital shaker. DNA was purified using the CTAB method (Ausubel et al., 1987). DNA was precipitated with isopropanol at −20°C overnight and then washed with 70% ethanol. After drying, the pellet was re‐suspended for the PCR assay in 25 μl of DNase‐free water.

The identity of citrus canker bacteria was verified using primers 2 and 3 (Hartung et al., 1993). PCR reactions were conducted at 25 μl reaction volume and PCR programs were conducted according to Cubero and Graham (2002).

### 2.5 Genotyping

Genotyping was performed on bacterial suspensions prepared for the whole strain collection (Table S1), plus reference strains, using a set of 14 microsatellites (MLVA‐14), mostly as reported previously (Pruvost et al., 2019). The MLVA‐14 scheme was supplemented with two additional PCR primer pairs, which respectively targeted the *copL* and *copD* genes, specific to each of the two copper resistance systems identified so far in *X. citri* pv*. citri* (Richard et al., 2017). For this, the primer pairs copL‐F (5′ CCGTGTCAGCCTCCTCACTTCTAC 3′)/copL‐R (5′ FAM‐CAGCGGCATGACATCCAGGCC 3′) and copD‐F (5′ CGACACGGATCACCCACGTCA 3′)/copD‐R (5′ NED‐TCTCCATCCGTCTCGCGCTCT 3′) were included in the multiplex PCR pools 1 and 2, respectively (Behlau et al., 2013; Richard et al., 2017). The controls in each plate were the strains LN002‐1 (IAPAR 306 in which the pLH201.1 plasmid hosting the *copLAB* system was introduced by conjugation) and LN173‐1 (IAPAR 306 in which the pLM199.1 plasmid hosting the *copABCD* system was introduced by conjugation). The number of tandem repeats (TR) at each locus was computed from fragment length for all assayed strains, using GeneMapper 4.0 (Applied Biosystems, Courtaboeuf, France) and was used as input data. Genetic diversity indices for MLVA‐14 data were calculated using the poppr 2.8.3 package (Kamvar et al., 2014) in R version 3.6.1 (https://www.R‐project.org/). Allelic richness (A) was computed using the rarefaction procedure for unequal sample sizes and the hierfstat 0.04–22 R package (Goudet, 2005). Private alleles were identified using poppr 2.9.3 (Kamvar et al., 2014). The algorithm recommended for MLVA data was used to produce minimum spanning networks, combining global optimal eBURST and Euclidean distances in PHYLOViZ v1.2 (Francisco et al., 2012). The population structure of *X. citri* pv. *citri* was then subject to the discriminant analysis of principal components (DAPC) using the adegenet 2.1.1. R package (Jombart, 2008; Jombart et al., 2010). This method is free of any assumption linked to a population genetic model (e.g., Hardy–Weinberg equilibrium or linkage equilibrium), which makes it suitable for analyzing datasets produced from predominantly clonal bacteria. Genetic differentiation (based on R_ST_) was computed using the genepop 1.1.3 package in R (Rousset, 2008). The occurrence of spatial structure was estimated using the global.rtest and related functions of the adegenet package.

A subset of strains selected on the basis of microsatellite data was further genotyped using 31 minisatellites (MLVA‐31). The aim was to compare them to a worldwide *X. citri* pv*. citri* strain collection of (http://www.biopred.net/MLVA/) and assign them to genetic lineages, which were delineated for this bacterium as described previously (Pruvost et al., 2014, 2015).

Drawing on minisatellite data, strains were assigned to genetic lineages and sub‐clusters, as previously reported (Leduc et al., 2015).

## 3 RESULTS

### 3.1 Surveyed areas and strain characterization

CBC was widely detected in most sampled regions in Saudi Arabia and Yemen (Figure 1a and Table S1) with the single exception of Al‐Madina region where citrus is grown commercially but no canker symptoms were found. All strains with a cultural morphology typical of *Xanthomonas* produced amplicons when assayed by PCR with the 2/3 primers (Hartung et al., 1993). This suggests that no strains of *X. citri* pv*. aurantifolii* were present in our Saudi and Yemeni strain collection. This finding was further confirmed by phenotypic assays (i.e., strains hydrolyzed gelatin and casein and were able to grow in the presence of 3% NaCl). Moreover, when microsatellite genotyping was performed on the whole strain collection (MLVA‐14), amplicons were produced for all strains at all targeted loci. This is typical of *X. citri* pv*. citri*, but unlike *X. citri* pv*. aurantifolii* (Bui Thi Ngoc, Vernière, Jarne, et al., 2009; Bui Thi Ngoc, Vernière, Vital, et al., 2009). Apart from the positive controls, not a single strain yielded PCR amplification with primers that target copper resistance genes (which are involved in the two known resistance systems in *X. citri* pv*. citri*). This suggests that they are susceptible to copper (a pesticide widely used for controlling CBC). Consistent with these results, none of 36 strains (selected on the basis of their genetic diversity—Table S1) grew on YPGA medium supplemented with copper. All assayed strains yielded a typical growth on the basal medium, thus phenotypically confirming them as copper‐susceptible.

### 3.2 Minisatellite‐based assignation of strains from the Arabian Peninsula to *X*. *citri* pv. *citri* lineages

**FIGURE 2:**
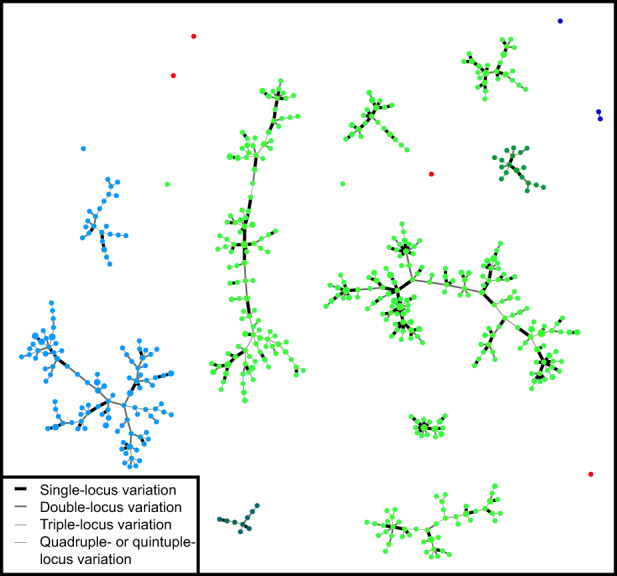
Minimum spanning tree from MLVA‐14 data showing the genetic diversity of *Xanthomonas citri* pv. *citri* in the Arabian Peninsula. All strains from distinct networks or singletons differed at ≥6 microsatellite loci. Dots represent haplotypes. Dot diameter and color are representative of the number of strains per haplotype, country, and host of isolation, respectively (light green: Saudi Arabia from Mexican lime; dark green: Saudi Arabia from other citrus; light blue: Yemen from Mexican lime; dark blue: Yemen from other citrus). Oman strains from a previous study (Vernière et al., 1998) are shown as red dots.

Microsatellite genotyping (MLVA‐14), which is known for its high discriminatory power (Bui Thi Ngoc, Vernière, Jarne, et al., 2009; Bui Thi Ngoc, Vernière, Vital, et al., 2009; Leduc et al., 2015; Vernière et al., 2014), identified 404 and 129 haplotypes among 563 and 164 strains from Saudi Arabia and Yemen, respectively. The minimum spanning tree built from this dataset (Figure 2) was used to identify a subset of 116 strains, representative of the revealed genetic diversity (93 originating from Saudi Arabia and 23 from Yemen). This sub‐collection was submitted to minisatellite genotyping (MLVA‐31) to situate strains from the present study in the known global diversity of *X. citri* pv. *citri* (Pruvost et al., 2014, 2015). The DAPC analysis assigned these strains to genetic lineages 1 (Yemen), 2 (Saudi Arabia), and 4 (both countries). All strains assigned to either lineage 1 or 2 (i.e., pathotype A strains) originated from several *Citrus* species, excluding Mexican lime. All strains originating from Mexican lime were assigned to lineage 4. The minimum spanning tree allowed placing lineage 4 strains to previously reported subclades (Pruvost et al., 2015). This confirmed the presence of subclades 4.1 and 4.2 in Saudi Arabia and subclades 4.2 and 4.4 in Yemen (Figure S1). Based on DAPC analysis, microsatellite data also confirmed the lineage 4 structure derived from minisatellite analysis (Figure S2).

Strains originating from the Arabian Peninsula and representative of each DAPC lineage (*n* = 36) were inoculated into Mexican lime and mandarin. All strains produced canker‐like lesions on Mexican lime. Conversely, only lineage 1 and 2 strains produced typical canker‐like lesions on mandarin, whereas lineage 4 strains produced small blister‐like lesions, surrounded by a water‐soaked area, typical of pathotype A and A* strains, respectively (Vernière et al., 1998). We found no obvious difference in the phenotype observed in response to inoculations between Saudi or Yemeni pathotype A* strains, which were assigned to distinct subclades using minisatellite analysis.

In summary, our results suggest that both countries host strains that are genetically related to pathotypes A and A*, but not A^w^. However, the latter was previously identified in neighboring Oman. Strains genetically related to pathotype A were detected in both countries, but assigned to distinct genetic lineages. All strains from Mexican lime were assigned to lineage 4, using minisatellite genotyping. Subclade 4.2 was detected in both countries and was overall prevalent in the analyzed strain collection. Each country hosted a distinct additional subclade (4.1 and 4.4 in Saudi Arabia and Yemen, respectively).

### 3.3 Genetic structure based on microsatellite data

At the country scale, multilocus Nei's index (H_exp_) computed from microsatellite data ranged from 0.55 (Yemen) to 0.71 (Saudi Arabia) and allelic richness (A) ranged from 8.0 (Yemen) to 10.5 (Saudi Arabia). This indicates high genotypic and allelic diversity in both countries (Table 1). Eighty‐seven and 24 private alleles (i.e., alleles only present in strains from a single country) were identified in the Saudi and Yemeni datasets, respectively. We used the microsatellite datasets to challenge the unexpected result that emerged from the minisatellite analysis, that is, only lineage 4 strains (pathotype A*) were identified on Mexican lime when both pathotypes A and A* were detected in Saudi Arabia and Yemen. Noticeably, not a single strain sampled from Mexican lime in Saudi Arabia (*n* = 535) and Yemen (*n* = 161) was found genetically related to pathotype A strains (Figure 2). This was clearly observed in the case of a Mexican lime block (JP2, *n* = 18—Table S1) that was the direct neighbor of a lemon block (JP1) where only lineage 2 strains (pathotype A) were detected.

**TABLE 1:** Genetic diversity parameters for *Xanthomonas citri* pv. *citri* strains originating from Saudi Arabia (*n* = 563) and Yemen (*n* = 164)

TR locus	Country	No. of alleles	Range of repeat numbers	H_exp_ ^a^	A^b^
XL1	S. Arabia	17	12–28	0.92	16.4
	Yemen	10	9–22	0.80	10.0
XL2	S. Arabia	12	17–37	0.80	10.6
	Yemen	7	8–37	0.34	7.0
XL3	S. Arabia	9	6–14	0.72	8.3
	Yemen	10	6–15	0.81	10.0
XL4	S. Arabia	17	6–24	0.85	13.7
	Yemen	11	9–20	0.82	11.0
XL5	S. Arabia	9	7–15	0.79	8.2
	Yemen	7	7–13	0.60	7.0
XL6	S. Arabia	33	8–66	0.88	24.9
	Yemen	17	14–32	0.92	17.0
XL7	S. Arabia	8	8–15	0.73	7.2
	Yemen	8	9–18	0.58	8.0
XL8	S. Arabia	6	3–8	0.53	4.3
	Yemen	6	4–9	0.23	6.0
XL9	S. Arabia	4	3–6	0.49	3.0
	Yemen	6	4–9	0.51	6.0
XL10	S. Arabia	9	4–12	0.76	7.7
	Yemen	6	5–12	0.43	6.0
XL11	S. Arabia	8	7–14	0.51	7.6
	Yemen	3	8–10	0.30	3.0
XL13	S. Arabia	17	8–25	0.86	14.9
	Yemen	6	8–13	0.55	6.0
XL14	S. Arabia	9	6–17	0.31	7.7
	Yemen	7	4–12	0.31	7.0
XL15	S. Arabia	17	7–26	0.75	12.5
	Yemen	8	5–13	0.56	8.0

^a^ Nei's index of genetic diversity (Nei, 1978).

^b^ Allelic richness computed by the rarefaction method.

**FIGURE 3:**
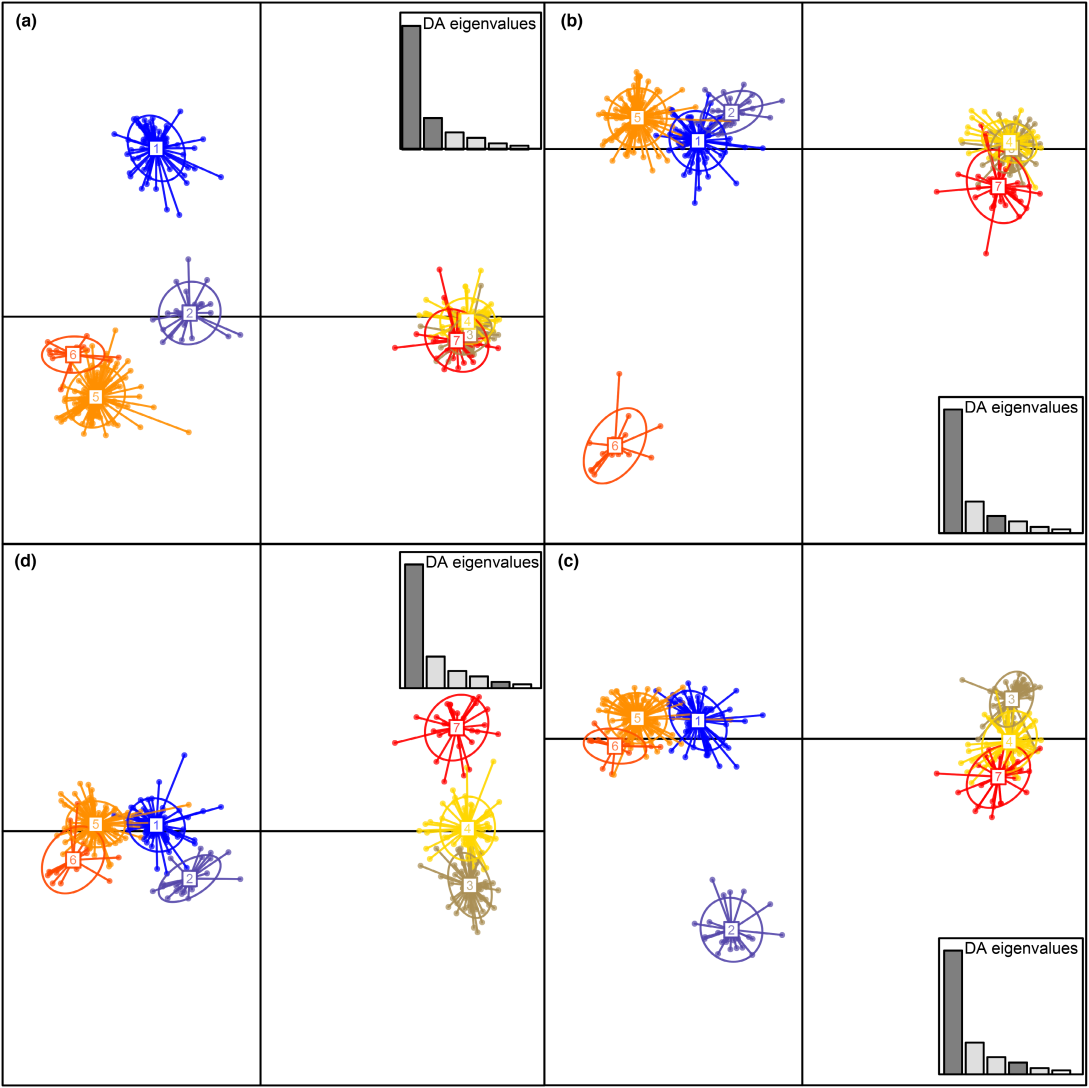
Genetic structure of *Xanthomonas citri* pv. *citri* subclade 4.2 originating from Saudi Arabia and Yemen based on the discriminant analysis of principal components (DAPC) of microsatellite data. Numbers and colors represent the seven genetic clusters retained from Bayesian information criterion (BIC) values. Clockwise: (a) scatterplot representing axes 1 and 2 of the DAPC; (b) scatterplot representing axes 1 and 3 of the DAPC; (c) scatterplot representing axes 1 and 4 of the DAPC; (d) scatterplot representing axes 1 and 5 of the DAPC.

We further focused our microsatellite analyses on the prevailing subclade 4.2. No global spatial structure (*p* = 0.094) was found. In contrast, a significant local structure (*p* = 0.001) was revealed, indicating strong genetic differences between strains sampled over short distances (i.e., a same site). All Yemenese strains (sampled from groves in the Al Hudaydah region) grouped in a single DAPC cluster (cluster #2) that contained no Saudi strain, a result that was consistent with the produced minimum spanning tree (Figure 3). Moreover, subclade 4.2 local populations from Yemen were strongly differentiated (*p* < 0.001) from all local 4.2 populations from Saudi Arabia, with R_ST_ values ranging from 0.23 to 0.94.

Saudi subclade 4.2 strains were assigned to six different DAPC clusters (Figure 3). Strains sampled from a same site were often assigned to several DAPC clusters, consistent with the detection of a significant local spatial structure. A significant genetic differentiation (*p* < 0.05) was observed among 95% (187/197) of remote (separated by ≥3 km) local population pairs. Analysis on local populations showed that 5% (5/98) of pairs of populations located in distinct regions were not differentiated (*p* > 0.05). The corresponding citrus groves were located in Jizan and Al Bahah regions at distances >300 km (Figure 1b). Consistently, some strains from these two regions grouped in the same DAPC clusters.

The microsatellite loci most frequently involved in differentiation among local populations were XL6 and to a lesser extent XL1, XL9, and XL13. Some strains originating from Saudi Arabia displayed a very large XL6 TR array (up to 66 repeats; Table 1). These markedly different allelic states at the XL6 locus were found for subclade 4.2 strains, which differed by up to ca. 40 repeats. Amplicons of various sizes were used for Sanger sequencing to determine the nature of the observed polymorphism. Four of these strains (including the one with 66 repeats) were analyzed by Sanger sequencing PCR amplicons. This confirmed that the large arrays are due to a higher number of repeats and not to DNA insertion in flanking regions and validated the number of TRs in the array that was determined from amplicon sizing (data not shown).

## 4 DISCUSSION

### 4.1 Genotyping data informs on the history and epidemiology of *X*. *citri* pv. *citri* in the Arabian Peninsula

We genotyped strains associated with the pioneer outbreaks of CBC in the Arabian Peninsula. The first outbreak reported in Yemen was based solely on visual observations. Therefore, the pathovar status (pv. *citri* vs. pv. *aurantifolii*) of this outbreak was at that time unresolved (Dimitman, 1984). Three strains isolated from Yemen in the 1980s, including one sampled during the very first outbreak reported in the country, were assigned to genetic lineage 1. They were further identified as pathotype A strains, based on pathogenicity assays. Minisatellite genotyping revealed that these strains were closely related. Their nearest relatives were strains from the Southwest Indian Ocean region and India (data not shown), which is consistent with the putative Indian origin of Yemeni strains presumed in the original report (Dimitman, 1984). All strains from our collection recently isolated from Mexican lime in Yemen were related to lineage 4. More specifically, lineage 4 subclade 4.2 (the prevalent subclade in Saudi Arabia) was identified in the Al Hudaydah region. Lineage 4 subclade 4.4 was identified in all sampled regions and is, therefore, the prevailing subclade in Yemen. Subclade 4.4 was previously reported in the Sistan‐Baluchistan region in Iran and sporadically in Mauritius (Pruvost et al., 2015, 2021). However, it should be pointed out that the main areas for sweet orange and mandarin cultivation in Yemen were not accessible for security reasons at the time of sampling. However, it is likely that pathotype A strains still occur in these areas of citrus cultivation, since no drastic eradication measures have been adopted (Dimitman, 1984). Indeed, there is no evidence that the pathogen will disappear given the prevailing disease management practices and there is no similar situation having occurred anywhere else.

In Saudi Arabia, strains from the first documented outbreak sampled in the Jizan area were assigned to genetic lineage 4 subclade 4.1 and further identified as pathotype A*, based on pathogenicity assays. The current hypothesis that *X. citri* pv. *citri* was introduced in Saudi Arabia from Yemen (Fadlallah, 1986) is clearly not supported by the genetic data. Interestingly, subclade 4.1 was only detected during the recent extensive survey from a single grove in exactly the same area, but not in other locations. This suggests that this subclade is unlikely to be widespread in citrus nurseries and not widely transmitted through the sale of plants for planting, a highly efficient pathway for the long‐distance spread of *X. citri* pv. *citri* (Leduc et al., 2015; Vernière et al., 2014); and supports that the prevailing environmental conditions in the region are unlikely to lead to long‐distance natural spread of the pathogen (Irey et al., 2006). Within groves, the pathogen is primarily spread through wind‐driven rain, overhead irrigation, and grove maintenance operations (Graham et al., 2004).

In the mid‐1980s, another outbreak in Saudi Arabia was reported in the Najran region (Fadlallah, 1986). The analysis of the available strains from this outbreak points to the second introduction of lineage 4 subclade 4.2. The analysis of recently sampled bacterial strains revealed the current prevalence of this subclade in Saudi Arabia.

The occurrence of pathotype A in Saudi Arabia was previously suggested (Al Saleh et al., 2014). However, Ibrahim et al. (2019) highlighted the need for an extensive molecular characterization of Saudi strains before conclusions can be drawn. Here, two groves of lemon and sweet orange, respectively, hosted lineage 2 strains, which were identified as pathotype A using pathogenicity assays. The present study shows the more extensive geographical distribution of this lineage, which was solely detected in the Indian subcontinent and West Africa prior to the present identification in the Arabian Peninsula (Leduc et al., 2015).

The occurrence of several *X. citri* pv. *citri* lineages and subclades in Saudi Arabia and Yemen suggests multiple introduction(s), very likely through contaminated plants for planting and/or propagative material. More specifically, the microsatellite DAPC cluster including the strains from the pioneering subclade 4.2 Saudi outbreak detected at the end of the 1980s was detected in all sampled Saudi regions, suggesting a role of citrus nurseries in regional spread of subclade 4.2. Subclade 4.2 was detected on plants for planting sampled in a single facility in the present study. Furthermore, a few pairs of local subclade 4.2 populations were genetically undifferentiated and geographically distant (>300 km). The natural spread of *X. citri* pv. *citri* has never been documented over such a distance even in association with extreme weather events (Irey et al., 2006). This suggests that the pathogen has likely been disseminated by contaminated citrus propagative material, a pathway that was associated with long‐distance spread of the pathogen in several countries (Leduc et al., 2015; Vernière et al., 2014).

Altogether, the presence of both pathotypes A and A* were ascertained in Saudi Arabia and Yemen. In the experimental design used herein, two citrus species, Mexican lime and mandarin, were used for pathogenicity tests. Therefore, it remains unknown whether some strain from the Arabian Peninsula displays a distinctive pathogenicity on other citrus species, a feature that was previously reported from Japan (Shiotani et al., 2007). In the present study, pathotype A strains were found to be clearly different, with lineage 1 and 2 identified from Yemen and Saudi Arabia, respectively, thus representing distinct introductions. We did not identify any pathotype A^w^ in our large strain collection, a group that has been reported from neighboring Oman (Gordon et al., 2015). However, sampling was conducted in two main areas and it cannot be entirely excluded that pathotype A^w^ strains are present in other areas in Saudi Arabia or Yemen. In contrast with pathotype A*, no major outbreak caused by pathotype A^w^ has been reported, neither in its probable area of origin (Indian subcontinent), nor in areas of fairly recent geographical expansion (Florida and Texas; Munoz Bodnar et al., 2017; Patane et al., 2019; Sun et al., 2004). Further research is required to decipher the factors associated with this apparent difference in epidemiological success.

### 4.2 Lineage 4 strains are highly prevalent on Mexican lime

Many crop pathogenic bacteria display a high host specialization, similar to bacteria that are pathogenic to vertebrates (Bull et al., 2010; Gilbert et al., 2012; Shaw et al., 2020). Indeed, bacterial pathogens spread naturally in agroecosystems, where its host abundance is high and its diversity is very low (if not null; McDonald & Stukenbrock, 2016; Thrall et al., 2007). Although the causes and consequences of host specialization have been theoretically studied, there is a need from experimental data comparing the performance and epidemiology of pathogenic strains varying in host specialization *in natura* (Barrett et al., 2009).

*Xanthomonas citri* pv. *citri* overall is clearly on the specialist side of the specialist‐generalist continuum that describes the pathogens' host range (Barrett et al., 2009; Graham et al., 2004). However, at the infrapathovar level, this pathogen still displays significant differences in host specialization (i.e., the *X. citri* pv. *citri* pathotypes). The present study represents the first extensive population analysis of *X. citri* pv. *citri* in two countries where both pathotypes A and A* occur. We focused on bacterial strains isolated from Mexican lime, a highly susceptible host species for all known pathotypes causing CBC (Gottwald et al., 2002). One of the main findings in the present study was that specialist strains of a crop bacterial pathogen suggested an ability to outcompete generalist strains *in natura*. Consistent with theoretical studies (Gandon, 2004; Leggett et al., 2013) and studies conducted on other organisms (Baumler & Fang, 2013; Morris & Moury, 2019), we suggest a fitness cost linked to wide host range pathotype A strains. Generalist bacterial haplotypes with a lower fitness are expected to be counter‐selected and may be ultimately removed from the population (Sheppard et al., 2018). The analysis of biological interactions in several ecosystems suggests that host–parasite specialization tends to be promoted by host abundance, which is clearly the case for agroecosystems (Vazquez et al., 2005). However, several other factors, such as cooperation or competition between bacterial actors through several mechanisms (e.g., bacteriocins, toxins, effectors exported by type 4 and 6 secretion systems…), involving other partners (e.g., bacteriophages, predating amoeba, the microbiota…) or other parameters (e.g., human activities and prevailing environmental conditions) can also modulate how specialist and generalist pathogens can successfully maintain in scenarios of co‐infection (Baldeweg et al., 2019; Bayer‐Santos et al., 2018; Gottwald et al., 2002; Leggett et al., 2013; Riley & Wertz, 2002; Sieiro et al., 2020; Souza et al., 2015; Vannier et al., 2019; Zhu et al., 2020). Therefore, further experiments under controlled conditions are required to assess virulence and/or transmission ability of specialized and generalist bacterial strains on Mexican lime and to determine the underlying mechanisms associated with potential differences. Indeed, the virulence of some pathogens that are able to infect multiple hosts can be host‐dependent (Leggett et al., 2013).

Consistent with the general concept that a precise characterization and surveillance of outbreak populations have clear fallout on disease control, the present study has implications for ACC management in Saudi Arabia (Bull & Koike, 2015; Woolhouse, 2002). First, the wide genetic diversity revealed in this paper is likely to complexify the intended use of bacteriophages for ACC control in Saudi Arabia (Ibrahim et al., 2017) and should orient its future development. Second, it suggests that resistance to copper compounds, widely used to control ACC worldwide, has not yet developed in the sampled regions. Indeed, our results suggest that copper sprays are still efficient but regular surveillance is required in future. Copper resistance in *X. citri* pv. *citri* was first reported in pathotype A strains from Argentina in the mid‐1990s (Canteros et al., 2017) and more recently in remote territories (Huang et al., 2021; Richard et al., 2016, 2017). Moreover, a recent genomic study revealed its presence in pathotype A* strains from Southeast Asia (Webster et al., 2020). Because of the risk of future emergence of copper‐resistant strains and the environmental issues caused by massive copper applications, research of alternative control strategies should be pursued. Third, the precise characterization of outbreak populations may allow a local optimization of the genetic composition of citrus blocks (including implementation of grafting‐on operations) in situations where only specialist strains occur. Finally, our study supported the role of citrus nurseries in the spread of at least a pathotype A* lineage in Saudi Arabia and underlines the need to strengthen (i) quarantine and certification schemes to produce pathogen‐free budwood for nurserymen, which may contribute to minimize unofficial budwood introductions and (ii) stricter surveillance of citrus nurseries in the region. Minimizing sources of primary inoculum and maximizing genetic drift through integrated pest management strategies have the ability to minimize the genetic diversity of *X. citri* pv. *citri*, constraint its microevolution through horizontal gene transfer or point mutations and avoid or at least delay the emergence of more invasive strains (Zhan et al., 2015).

## CONFLICT OF INTEREST

None declared.

## Supporting information


Figure S1

Figure S2

Table S1

Appendix S1

## Data Availability

The microsatellite data that support the findings of this study are openly available in the CIRAD dataverse at https://dataverse.cirad.fr/dataverse/pvbmt doi:10.18167/DVN1/CI05C6. Minisatellite data are available on the dedicated public database at http://www.biopred.net/mlva/.
