# Supplementary figures and images for: Molecular epidemiology of the citrus bacterial pathogen Xanthomonas citri pv. citri from the Arabian Peninsula reveals a complex structure of specialist and generalist strains
Source: Evol Appl. 2022 Aug 26;15(9):1423–35. doi: 10.1111/eva.13451 (PMC9488683; doi:10.1111/eva.13451)

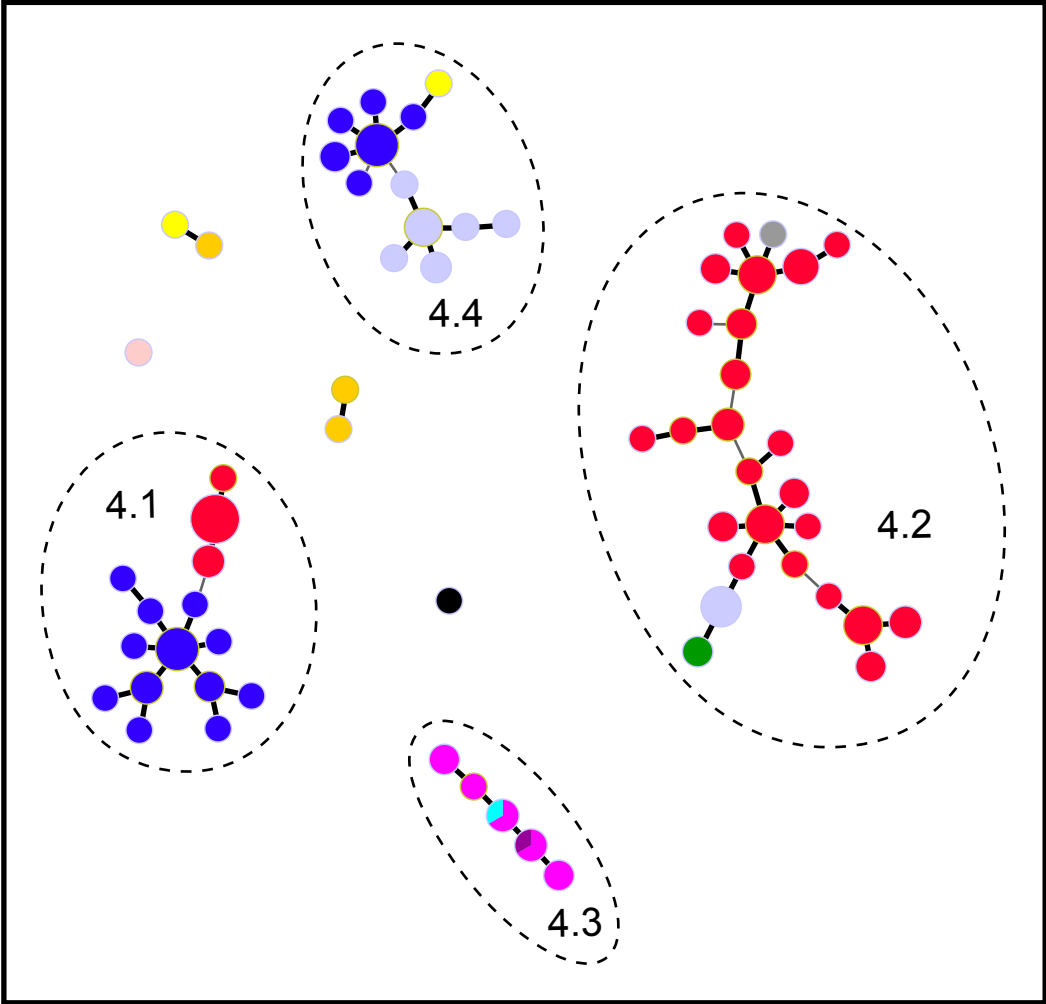

Supplement: Supplementary file 1 — Figure S1 [file EVA-15-1423-s003.pdf]

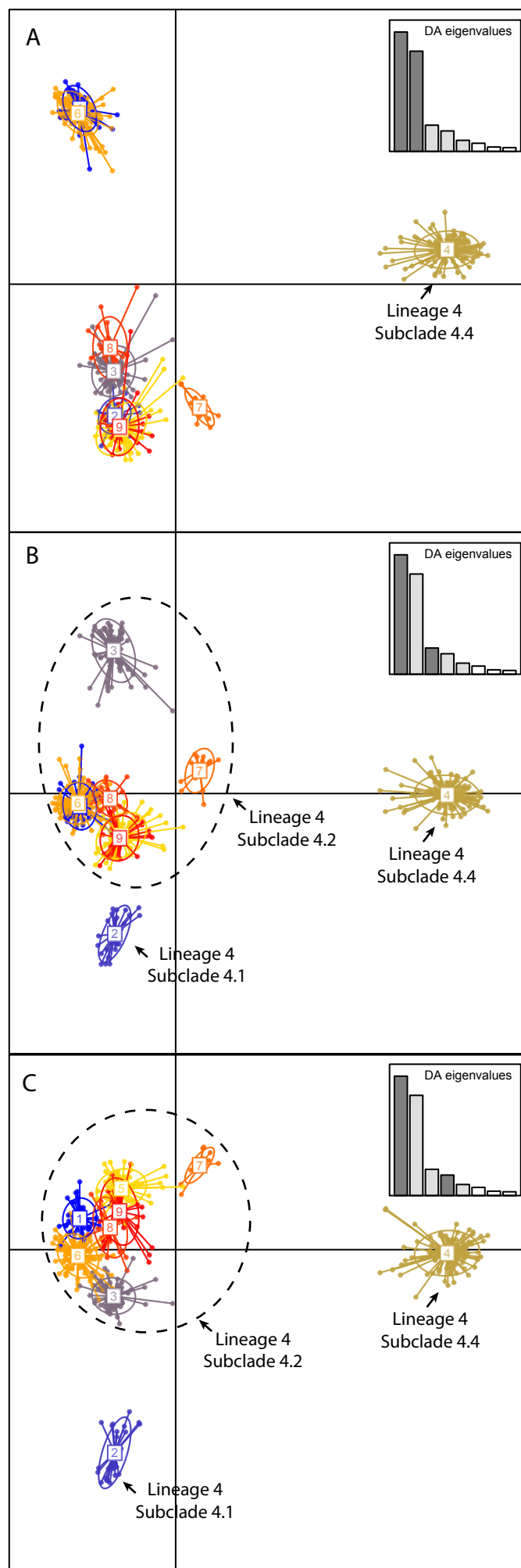

Supplement: Supplementary file 2 — Figure S2 [file EVA-15-1423-s004.pdf]
