# Supplementary material for: Molecular epidemiology of the citrus bacterial pathogen Xanthomonas citri pv. citri from the Arabian Peninsula reveals a complex structure of specialist and generalist strains
Source: Evol Appl. 2022 Aug 26;15(9):1423–35. doi: 10.1111/eva.13451 (PMC9488683; doi:10.1111/eva.13451)
Supplement: Supplementary file 3 — Table S1 [file EVA-15-1423-s002.docx]

# TABLE S1. *Xanthomonas* *citri* pv. *citri* strains from Saudi Arabia (n = 563) and YE (n = 164) used in this study

| No. | Strain | Population no. | Isolate ID | MLVA-14 haplotype | Year of isolation | Province | Plot | Host of isolation |
| --- | --- | --- | --- | --- | --- | --- | --- | --- |
|  | LQ036-02 | B12 | B12-1 | AS0001 | 2016 | SA^1^ - Al-Baha | Grove | Mexican Lime |
|  | LQ036-03 | B12 | B12-2 | AS0001 | 2016 | SA - Al-Baha | Grove | Mexican Lime |
|  | LR019-12 | B13 | B13-1 | AS0001 | 2016 | SA - Al-Baha | Grove | Mexican Lime |
|  | LQ010-03 | JP3 | JP3-1 | AS0002 | 2014 | SA – Jizan | Grove | Mexican Lime |
|  | LQ010-03A | JP3 | JP3-2 | AS0002 | 2014 | SA – Jizan | Grove | Mexican Lime |
|  | LQ010-03B | JP3 | JP3-3 | AS0002 | 2014 | SA – Jizan | Grove | Mexican Lime |
|  | LQ037-01a | B10 | B10-1 | AS0003 | 2016 | SA - Al-Baha | Grove | Mexican Lime |
|  | LQ037-01b | B10 | B10-2 | AS0003 | 2016 | SA - Al-Baha | Grove | Mexican Lime |
|  | LQ037-03 | B10 | B10-3 | AS0003 | 2016 | SA - Al-Baha | Grove | Mexican Lime |
|  | LR014-02 | B10 | B10-4 | AS0003 | 2016 | SA - Al-Baha | Grove | Mexican Lime |
|  | LR014-07 | B10 | B10-5 | AS0003 | 2016 | SA - Al-Baha | Grove | Mexican Lime |
|  | LR014-08 | B10 | B10-6 | AS0003 | 2016 | SA - Al-Baha | Grove | Mexican Lime |
|  | LR014-11 | B10 | B10-8 | AS0003 | 2016 | SA - Al-Baha | Grove | Mexican Lime |
|  | LQ021-03 | B4 | B4-1 | AS0004 | 2016 | SA - Al-Baha | Grove | Mexican Lime |
|  | LQ021-03A | B4 | B4-2 | AS0004 | 2016 | SA - Al-Baha | Grove | Mexican Lime |
|  | LQ021-03B | B4 | B4-3 | AS0004 | 2016 | SA - Al-Baha | Grove | Mexican Lime |
|  | LR012-02 | B4 | B4-4 | AS0004 | 2016 | SA - Al-Baha | Grove | Mexican Lime |
|  | LR012-03 | B4 | B4-5 | AS0004 | 2016 | SA - Al-Baha | Grove | Mexican Lime |
|  | LQ021-01 | B4 | B4-6 | AS0005 | 2016 | SA - Al-Baha | Grove | Mexican Lime |
|  | LQ021-01A | B4 | B4-7 | AS0005 | 2016 | SA - Al-Baha | Grove | Mexican Lime |
|  | LQ021-01B | B4 | B4-8 | AS0005 | 2016 | SA - Al-Baha | Grove | Mexican Lime |
|  | LQ019-01 | AB1 | ABP1-1 | AS0006 | 2014 | SA – Aseer | Grove | Mexican Lime |
|  | LQ019-03 | AB1 | ABP1-2 | AS0006 | 2014 | SA – Aseer | Grove | Mexican Lime |
|  | LQ019-03A | AB1 | ABP1-3 | AS0006 | 2014 | SA – Aseer | Grove | Mexican Lime |
|  | LQ019-03B | AB1 | ABP1-4 | AS0006 | 2014 | SA – Aseer | Grove | Mexican Lime |
|  | LR015-03 | AB1 | ABP1-5 | AS0006 | 2014 | SA – Aseer | Grove | Mexican Lime |
|  | LR015-13 | AB1 | ABP1-6 | AS0006 | 2014 | SA – Aseer | Grove | Mexican Lime |
|  | LQ010-01 | JP3 | JP3-4 | AS0007 | 2014 | SA – Jizan | Grove | Mexican Lime |
|  | LQ010-01A***** | JP3 | JP3-5 | AS0007 | 2014 | SA – Jizan | Grove | Mexican Lime |
|  | LQ010-01B | JP3 | JP3-6 | AS0007 | 2014 | SA – Jizan | Grove | Mexican Lime |
|  | LQ010-01C | JP3 | JP3-7 | AS0007 | 2014 | SA – Jizan | Grove | Mexican Lime |
|  | LQ012-03 | JP5 | JP5-1 | AS0008 | 2014 | SA – Jizan | Grove | Mexican Lime |
|  | LQ028-03 | JP5 | JP5-2 | AS0008 | 2014 | SA – Jizan | Grove | Mexican Lime |
|  | LR023-10 | JP5 | JP5-3 | AS0008 | 2014 | SA – Jizan | Grove | Mexican Lime |
|  | LQ034-04 | B14 | B14-1 | AS0009 | 2016 | SA - Al-Baha | Grove | Mexican Lime |
|  | LQ034-04A | B14 | B14-2 | AS0009 | 2016 | SA - Al-Baha | Grove | Mexican Lime |
|  | LQ034-04C | B14 | B14-3 | AS0009 | 2016 | SA - Al-Baha | Grove | Mexican Lime |
|  | LQ027-02 | YLP4 | YLP4-1 | AS0010 | 2014 | YE^2^- Lahj | Grove | Mexican Lime |
|  | LQ027-02A | YLP4 | YLP4-2 | AS0010 | 2014 | YE- Lahj | Grove | Mexican Lime |
|  | LQ027-02B | YLP4 | YLP4-3 | AS0010 | 2014 | YE- Lahj | Grove | Mexican Lime |
|  | JK002-16 | NA | JK002-16 | AS0011 | 1988 | SA | Grove | NA^3^ |
|  | JK002-19 | NA | JK002-19 | AS0011 | 1988 | SA | Grove | NA |
|  | LQ044-01 | B8 | B8-1 | AS0012 | 2016 | SA - Al-Baha | Grove | Mexican Lime |
|  | LQ044-01A | B8 | B8-2 | AS0012 | 2016 | SA - Al-Baha | Grove | Mexican Lime |
|  | LQ044-01B | B8 | B8-3 | AS0012 | 2016 | SA - Al-Baha | Grove | Mexican Lime |
|  | LQ044-01C | B8 | B8-4 | AS0012 | 2016 | SA - Al-Baha | Grove | Mexican Lime |
|  | LQ025-01 | YLP2 | YLP2-1 | AS0013 | 2014 | YE- Lahj | Grove | Mexican Lime |
|  | LQ025-04 | YLP2 | YLP2-2 | AS0013 | 2014 | YE- Lahj | Grove | Mexican Lime |
|  | LQ025-02 | YLP2 | YLP2-3 | AS0014 | 2014 | YE- Lahj | Grove | Mexican Lime |
|  | LQ023-02***** | YTP2 | YTP2-1 | AS0014 | 2014 | YE- Taiz | Grove | Mexican Lime |
|  | LQ027-01 | YLP4 | YLP4-4 | AS0015 | 2014 | YE- Lahj | Grove | Mexican Lime |
|  | LQ027-01A | YLP4 | YLP4-5 | AS0015 | 2014 | YE- Lahj | Grove | Mexican Lime |
|  | LQ027-01B | YLP4 | YLP4-6 | AS0015 | 2014 | YE- Lahj | Grove | Mexican Lime |
|  | LQ027-01C | YLP4 | YLP4-7 | AS0015 | 2014 | YE- Lahj | Grove | Mexican Lime |
|  | JK002-20 | NA | JK002-20 | AS0016 | 1988 | SA | Grove | NA |
|  | JK002-21 | NA | JK002-21 | AS0016 | 1988 | SA | Grove | NA |
|  | JK002-17 | NA | JK002-17 | AS0017 | 1988 | SA | Grove | NA |
|  | JK002-18 | NA | JK002-18 | AS0017 | 1988 | SA | Grove | NA |
|  | JK002-11 | NA | JK002-11 | AS0018 | 1988 | SA | Grove | NA |
|  | JK002-12 | NA | JK002-12 | AS0018 | 1988 | SA | Grove | NA |
|  | JK002-14 | NA | JK002-14 | AS0018 | 1988 | SA | Grove | NA |
|  | JK051 | NA | JK051 | AS0018 | 1988 | SA | Grove | NA |
|  | JK002-22 | NA | JK002-22 | AS0019 | 1988 | SA | Grove | NA |
|  | JK002-23 | NA | JK002-23 | AS0019 | 1988 | SA | Grove | NA |
|  | JK047 | NA | JK047 | AS0019 | 1988 | SA | Grove | NA |
|  | LQ043-01 | B7 | B7-1 | AS0020 | 2016 | SA - Al-Baha | Grove | Mexican Lime |
|  | LQ043-01A | B7 | B7-2 | AS0020 | 2016 | SA - Al-Baha | Grove | Mexican Lime |
|  | LQ043-01C | B7 | B7-3 | AS0020 | 2016 | SA - Al-Baha | Grove | Mexican Lime |
|  | LR022-05 | B7 | B7-4 | AS0020 | 2016 | SA - Al-Baha | Grove | Mexican Lime |
|  | LR022-06 | B7 | B7-5 | AS0020 | 2016 | SA - Al-Baha | Grove | Mexican Lime |
|  | LQ044-02 | B8 | B8-5 | AS0021 | 2016 | SA - Al-Baha | Grove | Mexican Lime |
|  | LQ044-02A | B8 | B8-6 | AS0021 | 2016 | SA - Al-Baha | Grove | Mexican Lime |
|  | LQ044-02B | B8 | B8-7 | AS0021 | 2016 | SA - Al-Baha | Grove | Mexican Lime |
|  | LQ034-03 | B14 | B14-4 | AS0022 | 2016 | SA - Al-Baha | Grove | Mexican Lime |
|  | LQ034-03A | B14 | B14-6 | AS0022 | 2016 | SA - Al-Baha | Grove | Mexican Lime |
|  | LQ034-03B | B14 | B14-7 | AS0022 | 2016 | SA - Al-Baha | Grove | Mexican Lime |
|  | LQ040-01 | B2 | B2-1 | AS0023 | 2016 | SA - Al-Baha | Grove | Mexican Lime |
|  | LQ040-01A | B2 | B2-2 | AS0023 | 2016 | SA - Al-Baha | Grove | Mexican Lime |
|  | LQ040-01B | B2 | B2-3 | AS0023 | 2016 | SA - Al-Baha | Grove | Mexican Lime |
|  | LQ040-03 | B2 | B2-4 | AS0024 | 2016 | SA - Al-Baha | Grove | Mexican Lime |
|  | LQ040-03A | B2 | B2-5 | AS0024 | 2016 | SA - Al-Baha | Grove | Mexican Lime |
|  | LQ040-03B | B2 | B2-6 | AS0024 | 2016 | SA - Al-Baha | Grove | Mexican Lime |
|  | LQ040-03C | B2 | B2-7 | AS0024 | 2016 | SA - Al-Baha | Grove | Mexican Lime |
|  | LR016-05 | B2 | B2-8 | AS0024 | 2016 | SA - Al-Baha | Grove | Mexican Lime |
|  | LQ045-01 | B9 | B9-1 | AS0025 | 2016 | SA - Al-Baha | Grove | Mexican Lime |
|  | LQ045-01A | B9 | B9-2 | AS0025 | 2016 | SA - Al-Baha | Grove | Mexican Lime |
|  | LQ045-01B | B9 | B9-3 | AS0025 | 2016 | SA - Al-Baha | Grove | Mexican Lime |
|  | LQ015-02***** | JP8 | JP8-1 | AS0026 | 2014 | SA – Jizan | Grove | Mexican Lime |
|  | LQ015-02A***** | JP8 | JP8-2 | AS0026 | 2014 | SA – Jizan | Grove | Mexican Lime |
|  | LQ015-02B***** | JP8 | JP8-3 | AS0026 | 2014 | SA – Jizan | Grove | Mexican Lime |
|  | LQ015-02C | JP8 | JP8-5 | AS0026 | 2014 | SA – Jizan | Grove | Mexican Lime |
|  | LQ023-03 | YTP2 | YTP2-2 | AS0027 | 2014 | YE- Taiz | Grove | Mexican Lime |
|  | LQ023-03A | YTP2 | YTP2-3 | AS0027 | 2014 | YE- Taiz | Grove | Mexican Lime |
|  | LQ023-03B***** | YTP2 | YTP2-4 | AS0027 | 2014 | YE- Taiz | Grove | Mexican Lime |
|  | LQ023-03C | YTP2 | YTP2-5 | AS0027 | 2014 | YE- Taiz | Grove | Mexican Lime |
|  | LQ029-02 | YHP1 | YHP1-1 | AS0028 | 2014 | YE- Al-Hudaydah | Grove | Mexican Lime |
|  | LQ029-02A | YHP1 | YhP1-2 | AS0028 | 2014 | YE- Al-Hudaydah | Grove | Mexican Lime |
|  | LQ029-02B | YHP1 | YHP1-3 | AS0028 | 2014 | YE- Al-Hudaydah | Grove | Mexican Lime |
|  | LQ029-02C | YHP1 | YHP1-4 | AS0028 | 2014 | YE- Al-Hudaydah | Grove | Mexican Lime |
|  | LQ030-01 | YHP2 | YHP2-1 | AS0029 | 2014 | YE- Al-Hudaydah | Grove | Mexican Lime |
|  | LQ030-01A | YHP2 | YHP2-2 | AS0029 | 2014 | YE- Al-Hudaydah | Grove | Mexican Lime |
|  | LQ030-01B | YHP2 | YHP2-3 | AS0029 | 2014 | YE- Al-Hudaydah | Grove | Mexican Lime |
|  | LQ030-01C | YHP2 | YHP2-4 | AS0029 | 2014 | YE- Al-Hudaydah | Grove | Mexican Lime |
|  | LQ030-02 | YHP2 | YHP2-5 | AS0030 | 2014 | YE- Al-Hudaydah | Grove | Mexican Lime |
|  | LQ030-02A | YHP2 | YHP2-6 | AS0030 | 2014 | YE- Al-Hudaydah | Grove | Mexican Lime |
|  | LQ030-02B | YHP2 | YHP2-7 | AS0030 | 2014 | YE- Al-Hudaydah | Grove | Mexican Lime |
|  | LQ030-02C | YHP2 | YHP2-8 | AS0030 | 2014 | YE- Al-Hudaydah | Grove | Mexican Lime |
|  | LR029-04 | YHP2 | YHP2-9 | AS0030 | 2014 | YE- Al-Hudaydah | Grove | Mexican Lime |
|  | LQ030-03 | YHP2 | YHP2-10 | AS0031 | 2014 | YE- Al-Hudaydah | Grove | Mexican Lime |
|  | LQ030-03A | YHP2 | YHP2-11 | AS0031 | 2014 | YE- Al-Hudaydah | Grove | Mexican Lime |
|  | LQ030-03B | YHP2 | YHP2-12 | AS0031 | 2014 | YE- Al-Hudaydah | Grove | Mexican Lime |
|  | LQ030-03C | YHP2 | YHP2-13 | AS0031 | 2014 | YE- Al-Hudaydah | Grove | Mexican Lime |
|  | LR033-19 | YHP3 | YHP3-1 | AS0031 | 2014 | YE- Al-Hudaydah | Grove | Mexican Lime |
|  | LQ028-01 | YLP5 | YLP5-1 | AS0032 | 2014 | YE- Lahj | Grove | Mexican Lime |
|  | LQ028-01A | YLP5 | YLP5-2 | AS0032 | 2014 | YE- Lahj | Grove | Mexican Lime |
|  | LQ028-01B | YLP5 | YLP5-3 | AS0032 | 2014 | YE- Lahj | Grove | Mexican Lime |
|  | LQ026-03***** | YLP3 | YLP3-1 | AS0033 | 2014 | YE- Lahj | Grove | Mexican Lime |
|  | LQ026-03A | YLP3 | YLP3-2 | AS0033 | 2014 | YE- Lahj | Grove | Mexican Lime |
|  | LQ026-03B | YLP3 | YLP3-3 | AS0033 | 2014 | YE- Lahj | Grove | Mexican Lime |
|  | LQ024-03 | YLP1 | YLP1-1 | AS0034 | 2014 | YE- Lahj | Grove | Mexican Lime |
|  | LQ024-03A | YLP1 | YLP1-2 | AS0034 | 2014 | YE- Lahj | Grove | Mexican Lime |
|  | LQ024-03B | YLP1 | YLP1-3 | AS0034 | 2014 | YE- Lahj | Grove | Mexican Lime |
|  | LQ045-03 | B9 | B9-4 | AS0035 | 2016 | SA - Al-Baha | Grove | Mexican Lime |
|  | LQ045-03A | B9 | B9-5 | AS0035 | 2016 | SA - Al-Baha | Grove | Mexican Lime |
|  | LQ045-03B | B9 | B9-6 | AS0035 | 2016 | SA - Al-Baha | Grove | Mexican Lime |
|  | LQ045-03C | B9 | B9-7 | AS0035 | 2016 | SA - Al-Baha | Grove | Mexican Lime |
|  | LQ033-01 | B11 | B11-1 | AS0036 | 2016 | SA - Al-Baha | Grove | Mexican Lime |
|  | LQ033-02A | B11 | B11-2 | AS0036 | 2016 | SA - Al-Baha | Grove | Mexican Lime |
|  | LQ040-02 | B2 | B2-9 | AS0037 | 2016 | SA - Al-Baha | Grove | Mexican Lime |
|  | LR016-01 | B2 | B2-10 | AS0037 | 2016 | SA - Al-Baha | Grove | Mexican Lime |
|  | LR016-03 | B2 | B2-11 | AS0037 | 2016 | SA - Al-Baha | Grove | Mexican Lime |
|  | LR016-04 | B2 | B2-12 | AS0037 | 2016 | SA - Al-Baha | Grove | Mexican Lime |
|  | LR016-09 | B2 | B2-13 | AS0037 | 2016 | SA - Al-Baha | Grove | Mexican Lime |
|  | LR016-11 | B2 | B2-14 | AS0037 | 2016 | SA - Al-Baha | Grove | Mexican Lime |
|  | LR016-12 | B2 | B2-15 | AS0037 | 2016 | SA - Al-Baha | Grove | Mexican Lime |
|  | LR016-13 | B2 | B2-16 | AS0037 | 2016 | SA - Al-Baha | Grove | Mexican Lime |
|  | LR016-21 | B2 | B2-17 | AS0037 | 2016 | SA - Al-Baha | Grove | Mexican Lime |
|  | LQ042-01 | B3 | B3-1 | AS0038 | 2016 | SA - Al-Baha | Grove | Mexican Lime |
|  | LQ020-03***** | ABP2 | ABP2-1 | AS0039 | 2015 | SA - Asser | Grove | Sweet Orange |
|  | LQ036-01 | B12 | B12-3 | AS0040 | 2016 | SA - Al-Baha | Grove | Mexican Lime |
|  | LR046-01 | B12 | B12-4 | AS0040 | 2016 | SA - Al-Baha | Grove | Mexican Lime |
|  | LQ012-02 | JP5 | JP5-4 | AS0041 | 2014 | SA - Jizan | Grove | Mexican Lime |
|  | LQ016-02 | JP9 | JP9-1 | AS0042 | 2014 | SA - Jizan | Grove | Mexican Lime |
|  | LQ041-03 | B5 | B5-1 | AS0043 | 2016 | SA - Al-Baha | Grove | Mexican Lime |
|  | LQ041-03A | B5 | B5-2 | AS0043 | 2016 | SA - Al-Baha | Grove | Mexican Lime |
|  | LQ041-03B | B5 | B5-3 | AS0043 | 2016 | SA - Al-Baha | Grove | Mexican Lime |
|  | LR012-06 | B4 | B4-9 | AS0044 | 2016 | SA - Al-Baha | Grove | Mexican Lime |
|  | LR012-13 | B4 | B4-10 | AS0044 | 2016 | SA - Al-Baha | Grove | Mexican Lime |
|  | LR012-14 | B4 | B4-11 | AS0044 | 2016 | SA - Al-Baha | Grove | Mexican Lime |
|  | LQ041-01 | B5 | B5-4 | AS0044 | 2016 | SA - Al-Baha | Grove | Mexican Lime |
|  | LR013-04 | B5 | B5-5 | AS0044 | 2016 | SA - Al-Baha | Grove | Mexican Lime |
|  | LR013-17 | B5 | B5-6 | AS0044 | 2016 | SA - Al-Baha | Grove | Mexican Lime |
|  | LR013-23 | B5 | B5-7 | AS0044 | 2016 | SA - Al-Baha | Grove | Mexican Lime |
|  | LQ041-02 | B5 | B5-8 | AS0045 | 2016 | SA - Al-Baha | Grove | Mexican Lime |
|  | LQ021-02 | B4 | B4-12 | AS0046 | 2016 | SA - Al-Baha | Grove | Mexican Lime |
|  | LQ037-02 | B10 | B10-9 | AS0047 | 2016 | SA - Al-Baha | Grove | Mexican Lime |
|  | LQ010-02 | JP3 | JP3-8 | AS0048 | 2014 | SA - Jizan | Grove | Mexican Lime |
|  | LQ013-02 | JP6 | JP6-1 | AS0049 | 2014 | SA - Jizan | Grove | Mexican Lime |
|  | LQ016-03 | JP9 | JP9-2 | AS0050 | 2014 | SA - Jizan | Grove | Mexican Lime |
|  | LQ019-02 | AB1 | ABP1-7 | AS0051 | 2014 | SA - Aseer | Grove | Mexican Lime |
|  | LQ018-03 | N | N1 | AS0052 | 2014 | SA - Aseer | Nursery | Mexican Lime |
|  | LR010-03 | N | N2 | AS0052 | 2014 | SA - Aseer | Nursery | Mexican Lime |
|  | LR010-07 | N | N3 | AS0052 | 2014 | SA - Aseer | Nursery | Mexican Lime |
|  | JM035-02 | NA | JM035-02 | AS0053 | NA | SA | Grove | NA |
|  | LQ018-01 | N | N4 | AS0054 | 2014 | SA - Aseer | Nursery | Mexican Lime |
|  | LR010-06 | N | N5 | AS0054 | 2014 | SA - Aseer | Nursery | Mexican Lime |
|  | LQ014-03B | JP7 | JP7-1 | AS0055 | 2014 | SA - Jizan | Grove | Mexican Lime |
|  | LQ014-03 | JP7 | JP7-2 | AS0056 | 2014 | SA - Jizan | Grove | Mexican Lime |
|  | LQ014-03C | JP7 | JP7-3 | AS0056 | 2014 | SA - Jizan | Grove | Mexican Lime |
|  | LQ014-03A | JP7 | JP7-4 | AS0057 | 2014 | SA - Jizan | Grove | Mexican Lime |
|  | LQ013-01 | JP6 | JP6-2 | AS0058 | 2014 | SA - Jizan | Grove | Mexican Lime |
|  | LQ034-01 | B14 | B14-8 | AS0059 | 2016 | SA - Al-Baha | Grove | Mexican Lime |
|  | LR025-15 | B14 | B14-9 | AS0059 | 2016 | SA - Al-Baha | Grove | Mexican Lime |
|  | LQ034-04B | B14 | B14-10 | AS0060 | 2016 | SA - Al-Baha | Grove | Mexican Lime |
|  | LQ016-01 | JP9 | JP9-3 | AS0061 | 2014 | SA - Jizan | Grove | Mexican Lime |
|  | JM035-01 | NA | JM035-01 | AS0062 | NA | SA | Grove | NA |
|  | LQ015-01 | JP8 | JP8-6 | AS0063 | 2014 | SA - Jizan | Grove | Mexican Lime |
|  | LR039-16 | JP9 | JP9-4 | AS0063 | 2014 | SA - Jizan | Grove | Mexican Lime |
|  | LQ043-02 | B7 | B7-6 | AS0064 | 2016 | SA - Al-Baha | Grove | Mexican Lime |
|  | LQ035-02 | B13 | B13-2 | AS0065 | 2016 | SA - Al-Baha | Grove | Mexican Lime |
|  | LR019-09 | B13 | B13-3 | AS0065 | 2016 | SA - Al-Baha | Grove | Mexican Lime |
|  | LQ031-03 | YHP3 | YHP3-2 | AS0066 | 2014 | YE- Al-Hudaydah | Grove | Mexican Lime |
|  | JK046 | NA | JK046 | AS0067 | 1988 | SA | Grove | NA |
|  | JK002-09 | NA | JK002-09 | AS0068 | 1988 | SA | Grove | NA |
|  | LQ025-03 | YLP2 | YLP2-4 | AS0069 | 2014 | YE- Lahj | Grove | Mexican Lime |
|  | JK002-10 | NA | JK002-10 | AS0070 | 1988 | SA | Grove | NA |
|  | JK050 | NA | JK050 | AS0071 | 1988 | SA | Grove | NA |
|  | JM035-04 | NA | JM035-04 | AS0072 | NA | SA | Grove | NA |
|  | JK049 | NA | JK049 | AS0073 | 1988 | SA | Grove | NA |
|  | JM035-03 | NA | JM035-03 | AS0074 | NA | SA | Grove | NA |
|  | JK002-13 | NA | JK002-13 | AS0075 | 1988 | SA | Grove | NA |
|  | JK048 | NA | JK048 | AS0076 | 1988 | SA | Grove | NA |
|  | LQ043-01B | B7 | B7-7 | AS0077 | 2016 | SA - Al-Baha | Grove | Mexican Lime |
|  | LQ029-03 | YHP1 | YHP1-5 | AS0078 | 2014 | YE- Al-Hudaydah | Grove | Mexican Lime |
|  | LQ039-02 | B1 | B1-1 | AS0079 | 2016 | SA - Al-Baha | Grove | Mexican Lime |
|  | LQ027-03 | YLP4 | YLP4-8 | AS0080 | 2014 | YE- Lahj | Grove | Mexican Lime |
|  | LQ011-02 | JP4 | JP4-1 | AS0081 | 2014 | SA - Jizan | Grove | Mexican Lime |
|  | LQ011-01 | JP4 | JP4-2 | AS0082 | 2014 | SA - Jizan | Grove | Mexican Lime |
|  | LQ034-02 | B14 | B14-11 | AS0083 | 2016 | SA - Al-Baha | Grove | Mexican Lime |
|  | LR025-16 | B14 | B14-12 | AS0083 | 2016 | SA - Al-Baha | Grove | Mexican Lime |
|  | LQ011-03 | JP4 | JP4-3 | AS0084 | 2014 | SA - Jizan | Grove | Mexican Lime |
|  | LQ032-02 | B6 | B6-1 | AS0085 | 2016 | SA - Al-Baha | Grove | Mexican Lime |
|  | LQ032-01 | B6 | B6-2 | AS0086 | 2016 | SA - Al-Baha | Grove | Mexican Lime |
|  | LR017-27 | B6 | B6-3 | AS0086 | 2016 | SA - Al-Baha | Grove | Mexican Lime |
|  | LQ022-03***** | YTP1 | YTP1-1 | AS0087 | 2014 | YE- Taiz | Grove | Mexican Lime |
|  | LQ029-01 | YHP1 | YHP1-6 | AS0088 | 2014 | YE- Al-Hudaydah | Grove | Mexican Lime |
|  | LR032-15 | YHP1 | YHP1-7 | AS0088 | 2014 | YE- Al-Hudaydah | Grove | Mexican Lime |
|  | LQ023-01***** | YTP2 | YTP2-6 | AS0089 | 2014 | YE- Taiz | Grove | Mexican Lime |
|  | LQ022-01 | YTP1 | YTP1-2 | AS0090 | 2014 | YE- Taiz | Grove | Mexican Lime |
|  | LQ022-02 | YTP1 | YTP1-3 | AS0091 | 2014 | YE- Taiz | Grove | Mexican Lime |
|  | LQ024-02 | YLP1 | YLP1-4 | AS0092 | 2014 | YE- Lahj | Grove | Mexican Lime |
|  | LQ031-02 | YHP3 | YHP3-3 | AS0093 | 2014 | YE- Al-Hudaydah | Grove | Mexican Lime |
|  | LQ028-02 | YLP5 | YLP5-4 | AS0094 | 2014 | YE- Lahj | Grove | Mexican Lime |
|  | LQ024-01 | YLP1 | YLP1-5 | AS0095 | 2014 | YE- Lahj | Grove | Mexican Lime |
|  | LQ045-02 | B9 | B9-8 | AS0096 | 2016 | SA - Al-Baha | Grove | Mexican Lime |
|  | LR018-24 | B9 | B9-9 | AS0096 | 2016 | SA - Al-Baha | Grove | Mexican Lime |
|  | LQ033-02B | B11 | B11-3 | AS0097 | 2016 | SA - Al-Baha | Grove | Mexican Lime |
|  | LQ043-03 | B7 | B7-8 | AS0097 | 2016 | SA - Al-Baha | Grove | Mexican Lime |
|  | LQ035-01 | B13 | B13-4 | AS0098 | 2016 | SA - Al-Baha | Grove | Mexican Lime |
|  | LR019-08 | B13 | B13-5 | AS0098 | 2016 | SA - Al-Baha | Grove | Mexican Lime |
|  | LQ035-03 | B13 | B13-6 | AS0099 | 2016 | SA - Al-Baha | Grove | Mexican Lime |
|  | LR019-01 | B13 | B13-7 | AS0099 | 2016 | SA - Al-Baha | Grove | Mexican Lime |
|  | LQ009-02 | JP2 | JP2-1 | AS0100 | 2014 | SA - Jizan | Grove | Mexican Lime |
|  | LQ014-02 | JP7 | JP7-5 | AS0101 | 2014 | SA - Jizan | Grove | Mexican Lime |
|  | LQ009-01 | JP2 | JP2-2 | AS0102 | 2014 | SA - Jizan | Grove | Mexican Lime |
|  | LQ013-03 | JP6 | JP6-3 | AS0103 | 2014 | SA - Jizan | Grove | Mexican Lime |
|  | LQ014-01 | JP7 | JP7-6 | AS0104 | 2014 | SA - Jizan | Grove | Mexican Lime |
|  | LQ017-02***** | JP10 | JP10-1 | AS0105 | 2014 | SA - Jizan | Grove | Mexican Lime |
|  | LQ017-01***** | JP10 | JP10-2 | AS0106 | 2014 | SA - Jizan | Grove | Mexican Lime |
|  | LQ008-03 | JP1 | JP1-1 | AS0107 | 2014 | SA - Jizan | Grove | Lemon |
|  | LQ008-02 | JP1 | JP1-2 | AS0108 | 2014 | SA - Jizan | Grove | Lemon |
|  | LQ008-01 | JP1 | JP1-3 | AS0109 | 2014 | SA - Jizan | Grove | Lemon |
|  | LR030-06 | JP1 | JP1-4 | AS0109 | 2014 | SA - Jizan | Grove | Lemon |
|  | LR035-06 | YTP1 | YTP1-4 | AS0135 | 2014 | YE- Taiz | Grove | Mexican Lime |
|  | LR029-19 | YHP2 | YHP2-14 | AS0489 | 2014 | YE- Al-Hudaydah | Grove | Mexican Lime |
|  | LQ009-03 | JP2 | JP2-3 | AS0608 | 2014 | SA - Jizan | Grove | Mexican Lime |
|  | LQ012-01 | JP5 | JP5-5 | AS0609 | 2014 | SA - Jizan | Grove | Mexican Lime |
|  | LR010-01 | N | N6 | AS0610 | 2014 | SA - Aseer | Nursery | Mexican Lime |
|  | LQ018-02 | N | N7 | AS0611 | 2014 | SA - Aseer | Nursery | Mexican Lime |
|  | LQ020-04 | ABP2 | ABP2-2 | AS0612 | 2015 | SA - Aseer | Grove | Sweet Orange |
|  | LR010-02 | N | N8 | AS0716 | 2014 | SA - Aseer | Nursery | Mexican Lime |
|  | LR010-04 | N | N9 | AS0717 | 2014 | SA - Aseer | Nursery | Mexican Lime |
|  | LR010-05 | N | N10 | AS0718 | 2014 | SA - Aseer | Nursery | Mexican Lime |
|  | LR010-08 | N | N11 | AS0719 | 2014 | SA - Aseer | Nursery | Mexican Lime |
|  | LR010-16 | N | N12 | AS0719 | 2014 | SA - Aseer | Nursery | Mexican Lime |
|  | LR010-09 | N | N13 | AS0720 | 2014 | SA - Aseer | Nursery | Mexican Lime |
|  | LR010-10 | N | N14 | AS0721 | 2014 | SA - Aseer | Nursery | Mexican Lime |
|  | LR010-11 | N | N15 | AS0721 | 2014 | SA - Aseer | Nursery | Mexican Lime |
|  | LR010-12 | N | N16 | AS0722 | 2014 | SA - Aseer | Nursery | Mexican Lime |
|  | LR010-13 | N | N17 | AS0723 | 2014 | SA - Aseer | Nursery | Mexican Lime |
|  | LR010-15 | N | N18 | AS0724 | 2014 | SA - Aseer | Nursery | Mexican Lime |
|  | LR010-17 | N | N19 | AS0724 | 2014 | SA - Aseer | Nursery | Mexican Lime |
|  | LR011-01 | B1 | B1-2 | AS0725 | 2016 | SA - Al-Baha | Grove | Mexican Lime |
|  | LR011-02 | B1 | B1-3 | AS0726 | 2016 | SA - Al-Baha | Grove | Mexican Lime |
|  | LR011-07 | B1 | B1-4 | AS0727 | 2016 | SA - Al-Baha | Grove | Mexican Lime |
|  | LR011-03***** | B1 | B1-5 | AS0728 | 2016 | SA - Al-Baha | Grove | Mexican Lime |
|  | LR011-04 | B1 | B1-6 | AS0729 | 2016 | SA - Al-Baha | Grove | Mexican Lime |
|  | LR011-05***** | B1 | B1-7 | AS0730 | 2016 | SA - Al-Baha | Grove | Mexican Lime |
|  | LR011-06***** | B1 | B1-8 | AS0731 | 2016 | SA - Al-Baha | Grove | Mexican Lime |
|  | LR011-08 | B1 | B1-9 | AS0732 | 2016 | SA - Al-Baha | Grove | Mexican Lime |
|  | LR011-09 | B1 | B1-10 | AS0733 | 2016 | SA - Al-Baha | Grove | Mexican Lime |
|  | LR011-10 | B1 | B1-11 | AS0734 | 2016 | SA - Al-Baha | Grove | Mexican Lime |
|  | LR011-11 | B1 | B1-12 | AS0735 | 2016 | SA - Al-Baha | Grove | Mexican Lime |
|  | LR011-13 | B1 | B1-13 | AS0736 | 2016 | SA - Al-Baha | Grove | Mexican Lime |
|  | LR011-14 | B1 | B1-14 | AS0737 | 2016 | SA - Al-Baha | Grove | Mexican Lime |
|  | LR011-15***** | B1 | B1-15 | AS0737 | 2016 | SA - Al-Baha | Grove | Mexican Lime |
|  | LR011-16 | B1 | B1-16 | AS0738 | 2016 | SA - Al-Baha | Grove | Mexican Lime |
|  | LR011-17 | B1 | B1-17 | AS0739 | 2016 | SA - Al-Baha | Grove | Mexican Lime |
|  | LR011-18 | B1 | B1-18 | AS0740 | 2016 | SA - Al-Baha | Grove | Mexican Lime |
|  | LR011-19 | B1 | B1-19 | AS0741 | 2016 | SA - Al-Baha | Grove | Mexican Lime |
|  | LR011-21 | B1 | B1-20 | AS0742 | 2016 | SA - Al-Baha | Grove | Mexican Lime |
|  | LR011-22 | B1 | B1-21 | AS0742 | 2016 | SA - Al-Baha | Grove | Mexican Lime |
|  | LR012-01 | B4 | B4-13 | AS0743 | 2016 | SA - Al-Baha | Grove | Mexican Lime |
|  | LR012-04 | B4 | B4-14 | AS0744 | 2016 | SA - Al-Baha | Grove | Mexican Lime |
|  | LR012-05 | B4 | B4-15 | AS0745 | 2016 | SA - Al-Baha | Grove | Mexican Lime |
|  | LR018-20 | B9 | B9-10 | AS0745 | 2016 | SA - Al-Baha | Grove | Mexican Lime |
|  | LR012-07 | B4 | B4-16 | AS0746 | 2016 | SA - Al-Baha | Grove | Mexican Lime |
|  | LR012-08 | B4 | B4-17 | AS0746 | 2016 | SA - Al-Baha | Grove | Mexican Lime |
|  | LR012-09 | B4 | B4-18 | AS0747 | 2016 | SA - Al-Baha | Grove | Mexican Lime |
|  | LR012-10 | B4 | B4-19 | AS0747 | 2016 | SA - Al-Baha | Grove | Mexican Lime |
|  | LR012-11 | B4 | B4-20 | AS0748 | 2016 | SA - Al-Baha | Grove | Mexican Lime |
|  | LR018-21 | B9 | B9-11 | AS0748 | 2016 | SA - Al-Baha | Grove | Mexican Lime |
|  | LR012-12 | B4 | B4-21 | AS0749 | 2016 | SA - Al-Baha | Grove | Mexican Lime |
|  | LR012-17 | B4 | B4-22 | AS0749 | 2016 | SA - Al-Baha | Grove | Mexican Lime |
|  | LR012-15A | B4 | B4-23 | AS0750 | 2016 | SA - Al-Baha | Grove | Mexican Lime |
|  | LR013-01 | B5 | B5-9 | AS0751 | 2016 | SA - Al-Baha | Grove | Mexican Lime |
|  | LR013-02 | B5 | B5-10 | AS0752 | 2016 | SA - Al-Baha | Grove | Mexican Lime |
|  | LR013-03 | B5 | B5-11 | AS0753 | 2016 | SA - Al-Baha | Grove | Mexican Lime |
|  | LR013-05 | B5 | B5-12 | AS0754 | 2016 | SA - Al-Baha | Grove | Mexican Lime |
|  | LR013-06 | B5 | B5-13 | AS0754 | 2016 | SA - Al-Baha | Grove | Mexican Lime |
|  | LR013-07 | B5 | B5-14 | AS0755 | 2016 | SA - Al-Baha | Grove | Mexican Lime |
|  | LR013-08 | B5 | B5-15 | AS0756 | 2016 | SA - Al-Baha | Grove | Mexican Lime |
|  | LR013-09 | B5 | B5-16 | AS0756 | 2016 | SA - Al-Baha | Grove | Mexican Lime |
|  | LR013-10 | B5 | B5-17 | AS0757 | 2016 | SA - Al-Baha | Grove | Mexican Lime |
|  | LR013-11 | B5 | B5-18 | AS0758 | 2016 | SA - Al-Baha | Grove | Mexican Lime |
|  | LR013-12 | B5 | B5-19 | AS0759 | 2016 | SA - Al-Baha | Grove | Mexican Lime |
|  | LR013-16 | B5 | B5-20 | AS0759 | 2016 | SA - Al-Baha | Grove | Mexican Lime |
|  | LR013-18 | B5 | B5-21 | AS0759 | 2016 | SA - Al-Baha | Grove | Mexican Lime |
|  | LR013-19 | B5 | B5-22 | AS0759 | 2016 | SA - Al-Baha | Grove | Mexican Lime |
|  | LR013-20 | B5 | B5-23 | AS0759 | 2016 | SA - Al-Baha | Grove | Mexican Lime |
|  | LR013-21 | B5 | B5-24 | AS0759 | 2016 | SA - Al-Baha | Grove | Mexican Lime |
|  | LR013-22 | B5 | B5-25 | AS0760 | 2016 | SA - Al-Baha | Grove | Mexican Lime |
|  | LR013-24 | B5 | B5-26 | AS0761 | 2016 | SA - Al-Baha | Grove | Mexican Lime |
|  | LR014-01 | B10 | B10-10 | AS0762 | 2016 | SA - Al-Baha | Grove | Mexican Lime |
|  | LR014-03 | B10 | B10-11 | AS0763 | 2016 | SA - Al-Baha | Grove | Mexican Lime |
|  | LR014-04 | B10 | B10-12 | AS0763 | 2016 | SA - Al-Baha | Grove | Mexican Lime |
|  | LR014-05 | B10 | B10-13 | AS0764 | 2016 | SA - Al-Baha | Grove | Mexican Lime |
|  | LR014-09 | B10 | B10-14 | AS0764 | 2016 | SA - Al-Baha | Grove | Mexican Lime |
|  | LR014-06 | B10 | B10-15 | AS0765 | 2016 | SA - Al-Baha | Grove | Mexican Lime |
|  | LR014-10 | B10 | B10-16 | AS0765 | 2016 | SA - Al-Baha | Grove | Mexican Lime |
|  | LR014-12 | B10 | B10-17 | AS0766 | 2016 | SA - Al-Baha | Grove | Mexican Lime |
|  | LR014-13 | B10 | B10-18 | AS0767 | 2016 | SA - Al-Baha | Grove | Mexican Lime |
|  | LR014-14 | B10 | B10-19 | AS0768 | 2016 | SA - Al-Baha | Grove | Mexican Lime |
|  | LR014-15 | B10 | B10-20 | AS0769 | 2016 | SA - Al-Baha | Grove | Mexican Lime |
|  | LR014-16 | B10 | B10-21 | AS0770 | 2016 | SA - Al-Baha | Grove | Mexican Lime |
|  | LR015-01 | AB1 | ABP1-8 | AS0771 | 2014 | SA - Aseer | Grove | Mexican Lime |
|  | LR015-02 | AB1 | ABP1-9 | AS0772 | 2014 | SA - Aseer | Grove | Mexican Lime |
|  | LR015-04 | AB1 | ABP1-10 | AS0773 | 2014 | SA - Aseer | Grove | Mexican Lime |
|  | LR015-05 | AB1 | ABP1-11 | AS0774 | 2014 | SA - Aseer | Grove | Mexican Lime |
|  | LR015-06 | AB1 | ABP1-12 | AS0775 | 2014 | SA - Aseer | Grove | Mexican Lime |
|  | LR015-07 | AB1 | ABP1-13 | AS0776 | 2014 | SA - Aseer | Grove | Mexican Lime |
|  | LR015-08 | AB1 | ABP1-14 | AS0777 | 2014 | SA - Aseer | Grove | Mexican Lime |
|  | LR015-09 | AB1 | ABP1-16 | AS0778 | 2014 | SA - Aseer | Grove | Mexican Lime |
|  | LR015-10 | AB1 | ABP1-17 | AS0779 | 2014 | SA - Aseer | Grove | Mexican Lime |
|  | LR015-11 | AB1 | ABP1-18 | AS0780 | 2014 | SA - Aseer | Grove | Mexican Lime |
|  | LR015-12 | AB1 | ABP1-19 | AS0781 | 2014 | SA - Aseer | Grove | Mexican Lime |
|  | LR016-06 | B2 | B2-18 | AS0782 | 2016 | SA - Al-Baha | Grove | Mexican Lime |
|  | LR016-16 | B2 | B2-19 | AS0782 | 2016 | SA - Al-Baha | Grove | Mexican Lime |
|  | LR016-08 | B2 | B2-20 | AS0783 | 2016 | SA - Al-Baha | Grove | Mexican Lime |
|  | LR016-10 | B2 | B2-21 | AS0784 | 2016 | SA - Al-Baha | Grove | Mexican Lime |
|  | LR016-14 | B2 | B2-22 | AS0785 | 2016 | SA - Al-Baha | Grove | Mexican Lime |
|  | LR016-15 | B2 | B2-23 | AS0786 | 2016 | SA - Al-Baha | Grove | Mexican Lime |
|  | LR016-17 | B2 | B2-24 | AS0787 | 2016 | SA - Al-Baha | Grove | Mexican Lime |
|  | LR016-18 | B2 | B2-25 | AS0788 | 2016 | SA - Al-Baha | Grove | Mexican Lime |
|  | LR017-01 | B6 | B6-4 | AS0789 | 2016 | SA - Al-Baha | Grove | Mexican Lime |
|  | LR017-02 | B6 | B6-5 | AS0790 | 2016 | SA - Al-Baha | Grove | Mexican Lime |
|  | LR017-04 | B6 | B6-6 | AS0790 | 2016 | SA - Al-Baha | Grove | Mexican Lime |
|  | LR017-03 | B6 | B6-7 | AS0791 | 2016 | SA - Al-Baha | Grove | Mexican Lime |
|  | LR017-05 | B6 | B6-8 | AS0792 | 2016 | SA - Al-Baha | Grove | Mexican Lime |
|  | LR017-06 | B6 | B6-9 | AS0793 | 2016 | SA - Al-Baha | Grove | Mexican Lime |
|  | LR017-07 | B6 | B6-10 | AS0793 | 2016 | SA - Al-Baha | Grove | Mexican Lime |
|  | LR017-16 | B6 | B6-13 | AS0793 | 2016 | SA - Al-Baha | Grove | Mexican Lime |
|  | LR017-08 | B6 | B6-14 | AS0794 | 2016 | SA - Al-Baha | Grove | Mexican Lime |
|  | LR017-10 | B6 | B6-15 | AS0794 | 2016 | SA - Al-Baha | Grove | Mexican Lime |
|  | LR017-09 | B6 | B6-16 | AS0795 | 2016 | SA - Al-Baha | Grove | Mexican Lime |
|  | LR017-11 | B6 | B6-17 | AS0796 | 2016 | SA - Al-Baha | Grove | Mexican Lime |
|  | LR017-12 | B6 | B6-18 | AS0797 | 2016 | SA - Al-Baha | Grove | Mexican Lime |
|  | LR017-13 | B6 | B6-19 | AS0798 | 2016 | SA - Al-Baha | Grove | Mexican Lime |
|  | LR017-15 | B6 | B6-20 | AS0799 | 2016 | SA - Al-Baha | Grove | Mexican Lime |
|  | LR017-18 | B6 | B6-21 | AS0800 | 2016 | SA - Al-Baha | Grove | Mexican Lime |
|  | LR017-19 | B6 | B6-22 | AS0800 | 2016 | SA - Al-Baha | Grove | Mexican Lime |
|  | LR017-20 | B6 | B6-23 | AS0801 | 2016 | SA - Al-Baha | Grove | Mexican Lime |
|  | LR017-21 | B6 | B6-24 | AS0802 | 2016 | SA - Al-Baha | Grove | Mexican Lime |
|  | LR017-22 | B6 | B6-25 | AS0803 | 2016 | SA - Al-Baha | Grove | Mexican Lime |
|  | LR017-23 | B6 | B6-26 | AS0804 | 2016 | SA - Al-Baha | Grove | Mexican Lime |
|  | LR017-24 | B6 | B6-27 | AS0805 | 2016 | SA - Al-Baha | Grove | Mexican Lime |
|  | LR017-25 | B6 | B6-28 | AS0806 | 2016 | SA - Al-Baha | Grove | Mexican Lime |
|  | LR017-26 | B6 | B6-29 | AS0807 | 2016 | SA - Al-Baha | Grove | Mexican Lime |
|  | LR017-28 | B6 | B6-30 | AS0808 | 2016 | SA - Al-Baha | Grove | Mexican Lime |
|  | LR017-29 | B6 | B6-31 | AS0809 | 2016 | SA - Al-Baha | Grove | Mexican Lime |
|  | LR017-30 | B6 | B6-32 | AS0810 | 2016 | SA - Al-Baha | Grove | Mexican Lime |
|  | LR018-01 | B9 | B9-12 | AS0811 | 2016 | SA - Al-Baha | Grove | Mexican Lime |
|  | LR018-16 | B9 | B9-13 | AS0811 | 2016 | SA - Al-Baha | Grove | Mexican Lime |
|  | LR018-02 | B9 | B9-14 | AS0812 | 2016 | SA - Al-Baha | Grove | Mexican Lime |
|  | LR018-03 | B9 | B9-15 | AS0813 | 2016 | SA - Al-Baha | Grove | Mexican Lime |
|  | LR018-04 | B9 | B9-16 | AS0814 | 2016 | SA - Al-Baha | Grove | Mexican Lime |
|  | LR019-04 | B13 | B13-8 | AS0815 | 2016 | SA - Al-Baha | Grove | Mexican Lime |
|  | LR019-05 | B13 | B13-9 | AS0815 | 2016 | SA - Al-Baha | Grove | Mexican Lime |
|  | LR018-05 | B9 | B9-17 | AS0815 | 2016 | SA - Al-Baha | Grove | Mexican Lime |
|  | LR018-06 | B9 | B9-18 | AS0816 | 2016 | SA - Al-Baha | Grove | Mexican Lime |
|  | LR018-07 | B9 | B9-19 | AS0817 | 2016 | SA - Al-Baha | Grove | Mexican Lime |
|  | LR018-08 | B9 | B9-20 | AS0818 | 2016 | SA - Al-Baha | Grove | Mexican Lime |
|  | LR018-09 | B9 | B9-21 | AS0819 | 2016 | SA - Al-Baha | Grove | Mexican Lime |
|  | LR018-11 | B9 | B9-22 | AS0820 | 2016 | SA - Al-Baha | Grove | Mexican Lime |
|  | LR018-17 | B9 | B9-23 | AS0820 | 2016 | SA - Al-Baha | Grove | Mexican Lime |
|  | LR018-12 | B9 | B9-24 | AS0821 | 2016 | SA - Al-Baha | Grove | Mexican Lime |
|  | LR018-13 | B9 | B9-25 | AS0822 | 2016 | SA - Al-Baha | Grove | Mexican Lime |
|  | LR018-14 | B9 | B9-26 | AS0823 | 2016 | SA - Al-Baha | Grove | Mexican Lime |
|  | LR018-15 | B9 | B9-27 | AS0824 | 2016 | SA - Al-Baha | Grove | Mexican Lime |
|  | LR019-02 | B13 | B13-10 | AS0825 | 2016 | SA - Al-Baha | Grove | Mexican Lime |
|  | LR019-03 | B13 | B13-11 | AS0826 | 2016 | SA - Al-Baha | Grove | Mexican Lime |
|  | LR019-06 | B13 | B13-15 | AS0827 | 2016 | SA - Al-Baha | Grove | Mexican Lime |
|  | LR019-07 | B13 | B13-16 | AS0828 | 2016 | SA - Al-Baha | Grove | Mexican Lime |
|  | LR019-11 | B13 | B13-19 | AS0829 | 2016 | SA - Al-Baha | Grove | Mexican Lime |
|  | LR020-01***** | JP3 | JP3-9 | AS0830 | 2014 | SA - Jizan | Grove | Mexican Lime |
|  | LR020-02***** | JP3 | JP3-10 | AS0831 | 2014 | SA - Jizan | Grove | Mexican Lime |
|  | LR020-03 | JP3 | JP3-11 | AS0832 | 2014 | SA - Jizan | Grove | Mexican Lime |
|  | LR020-04 | JP3 | JP3-12 | AS0833 | 2014 | SA - Jizan | Grove | Mexican Lime |
|  | LR020-10 | JP3 | JP3-13 | AS0833 | 2014 | SA - Jizan | Grove | Mexican Lime |
|  | LR020-20 | JP3 | JP3-14 | AS0833 | 2014 | SA - Jizan | Grove | Mexican Lime |
|  | LR020-05 | JP3 | JP3-15 | AS0834 | 2014 | SA - Jizan | Grove | Mexican Lime |
|  | LR020-06***** | JP3 | JP3-16 | AS0835 | 2014 | SA - Jizan | Grove | Mexican Lime |
|  | LR020-16 | JP3 | JP3-17 | AS0835 | 2014 | SA - Jizan | Grove | Mexican Lime |
|  | LR020-07 | JP3 | JP3-18 | AS0836 | 2014 | SA - Jizan | Grove | Mexican Lime |
|  | LR020-08 | JP3 | JP3-19 | AS0837 | 2014 | SA - Jizan | Grove | Mexican Lime |
|  | LR020-09 | JP3 | JP3-20 | AS0838 | 2014 | SA - Jizan | Grove | Mexican Lime |
|  | LR020-11 | JP3 | JP3-21 | AS0839 | 2014 | SA - Jizan | Grove | Mexican Lime |
|  | LR020-12 | JP3 | JP3-22 | AS0840 | 2014 | SA - Jizan | Grove | Mexican Lime |
|  | LR020-13 | JP3 | JP3-23 | AS0841 | 2014 | SA - Jizan | Grove | Mexican Lime |
|  | LR020-14 | JP3 | JP3-24 | AS0842 | 2014 | SA - Jizan | Grove | Mexican Lime |
|  | LR020-21 | JP3 | JP3-25 | AS0842 | 2014 | SA - Jizan | Grove | Mexican Lime |
|  | LR020-15 | JP3 | JP3-26 | AS0843 | 2014 | SA - Jizan | Grove | Mexican Lime |
|  | LR020-17 | JP3 | JP3-27 | AS0844 | 2014 | SA - Jizan | Grove | Mexican Lime |
|  | LR020-18 | JP3 | JP3-28 | AS0845 | 2014 | SA - Jizan | Grove | Mexican Lime |
|  | LR020-19 | JP3 | JP3-29 | AS0846 | 2014 | SA - Jizan | Grove | Mexican Lime |
|  | LR021-01 | B3 | B3-2 | AS0847 | 2016 | SA - Al-Baha | Grove | Mexican Lime |
|  | LR021-02 | B3 | B3-3 | AS0847 | 2016 | SA - Al-Baha | Grove | Mexican Lime |
|  | LR021-04 | B3 | B3-4 | AS0847 | 2016 | SA - Al-Baha | Grove | Mexican Lime |
|  | LR021-03 | B3 | B3-5 | AS0848 | 2016 | SA - Al-Baha | Grove | Mexican Lime |
|  | LR021-05 | B3 | B3-6 | AS0849 | 2016 | SA - Al-Baha | Grove | Mexican Lime |
|  | LR021-06 | B3 | B3-7 | AS0850 | 2016 | SA - Al-Baha | Grove | Mexican Lime |
|  | LR022-01 | B7 | B7-9 | AS0851 | 2016 | SA - Al-Baha | Grove | Mexican Lime |
|  | LR022-07 | B7 | B7-10 | AS0851 | 2016 | SA - Al-Baha | Grove | Mexican Lime |
|  | LR022-02 | B7 | B7-11 | AS0852 | 2016 | SA - Al-Baha | Grove | Mexican Lime |
|  | LR022-11 | B7 | B7-12 | AS0852 | 2016 | SA - Al-Baha | Grove | Mexican Lime |
|  | LR022-03 | B7 | B7-13 | AS0853 | 2016 | SA - Al-Baha | Grove | Mexican Lime |
|  | LR022-04 | B7 | B7-14 | AS0854 | 2016 | SA - Al-Baha | Grove | Mexican Lime |
|  | LR022-16 | B7 | B7-15 | AS0854 | 2016 | SA - Al-Baha | Grove | Mexican Lime |
|  | LR022-19 | B7 | B7-16 | AS0854 | 2016 | SA - Al-Baha | Grove | Mexican Lime |
|  | LR022-08 | B7 | B7-17 | AS0855 | 2016 | SA - Al-Baha | Grove | Mexican Lime |
|  | LR022-09 | B7 | B7-18 | AS0856 | 2016 | SA - Al-Baha | Grove | Mexican Lime |
|  | LR022-12 | B7 | B7-19 | AS0856 | 2016 | SA - Al-Baha | Grove | Mexican Lime |
|  | LR022-10 | B7 | B7-20 | AS0857 | 2016 | SA - Al-Baha | Grove | Mexican Lime |
|  | LR022-13 | B7 | B7-21 | AS0858 | 2016 | SA - Al-Baha | Grove | Mexican Lime |
|  | LR022-14 | B7 | B7-22 | AS0859 | 2016 | SA - Al-Baha | Grove | Mexican Lime |
|  | LR022-15 | B7 | B7-23 | AS0860 | 2016 | SA - Al-Baha | Grove | Mexican Lime |
|  | LR022-17 | B7 | B7-24 | AS0861 | 2016 | SA - Al-Baha | Grove | Mexican Lime |
|  | LR022-18 | B7 | B7-25 | AS0862 | 2016 | SA - Al-Baha | Grove | Mexican Lime |
|  | LR022-20 | B7 | B7-26 | AS0863 | 2016 | SA - Al-Baha | Grove | Mexican Lime |
|  | LR023-01 | JP5 | JP5-6 | AS0864 | 2014 | SA - Jizan | Grove | Mexican Lime |
|  | LR023-02 | JP5 | JP5-7 | AS0865 | 2014 | SA - Jizan | Grove | Mexican Lime |
|  | LR023-04 | JP5 | JP5-8 | AS0865 | 2014 | SA - Jizan | Grove | Mexican Lime |
|  | LR023-05 | JP5 | JP5-9 | AS0865 | 2014 | SA - Jizan | Grove | Mexican Lime |
|  | LR023-06 | JP5 | JP5-10 | AS0866 | 2014 | SA - Jizan | Grove | Mexican Lime |
|  | LR023-07 | JP5 | JP5-11 | AS0867 | 2014 | SA - Jizan | Grove | Mexican Lime |
|  | LR023-08 | JP5 | JP5-12 | AS0868 | 2014 | SA - Jizan | Grove | Mexican Lime |
|  | LR023-09 | JP5 | JP5-13 | AS0869 | 2014 | SA - Jizan | Grove | Mexican Lime |
|  | LR023-11 | JP5 | JP5-14 | AS0870 | 2014 | SA - Jizan | Grove | Mexican Lime |
|  | LR027-02 | JP4 | JP4-4 | AS0871 | 2014 | SA - Jizan | Grove | Mexican Lime |
|  | LR027-03 | JP4 | JP4-5 | AS0871 | 2014 | SA - Jizan | Grove | Mexican Lime |
|  | LR027-16 | JP4 | JP4-6 | AS0871 | 2014 | SA - Jizan | Grove | Mexican Lime |
|  | LR023-12 | JP5 | JP5-15 | AS0871 | 2014 | SA - Jizan | Grove | Mexican Lime |
|  | LR023-13 | JP5 | JP5-16 | AS0872 | 2014 | SA - Jizan | Grove | Mexican Lime |
|  | LR023-14 | JP5 | JP5-17 | AS0873 | 2014 | SA - Jizan | Grove | Mexican Lime |
|  | LR023-03 | JP5 | JP5-18 | AS0874 | 2014 | SA - Jizan | Grove | Mexican Lime |
|  | LR023-15 | JP5 | JP5-19 | AS0874 | 2014 | SA - Jizan | Grove | Mexican Lime |
|  | LR023-16 | JP5 | JP5-20 | AS0875 | 2014 | SA - Jizan | Grove | Mexican Lime |
|  | LR024-01 | ABP2 | ABP2-4 | AS0876 | 2015 | SA - Aseer | Grove | Sweet Orange |
|  | LR024-03***** | ABP2 | ABP2-7 | AS0878 | 2015 | SA - Aseer | Grove | Sweet Orange |
|  | LR024-04 | ABP2 | ABP2-8 | AS0879 | 2015 | SA - Aseer | Grove | Sweet Orange |
|  | LR024-05***** | ABP2 | ABP2-9 | AS0880 | 2015 | SA - Aseer | Grove | Sweet Orange |
|  | LR024-08 | ABP2 | ABP2-10 | AS0881 | 2015 | SA - Aseer | Grove | Sweet Orange |
|  | LR025-01 | B14 | B14-13 | AS0882 | 2016 | SA - Al-Baha | Grove | Mexican Lime |
|  | LR025-02 | B14 | B14-14 | AS0884 | 2016 | SA - Al-Baha | Grove | Mexican Lime |
|  | LR025-03 | B14 | B14-15 | AS0885 | 2016 | SA - Al-Baha | Grove | Mexican Lime |
|  | LR025-05 | B14 | B14-16 | AS0886 | 2016 | SA - Al-Baha | Grove | Mexican Lime |
|  | LR025-06 | B14 | B14-17 | AS0887 | 2016 | SA - Al-Baha | Grove | Mexican Lime |
|  | LR025-07 | B14 | B14-18 | AS0888 | 2016 | SA - Al-Baha | Grove | Mexican Lime |
|  | LR025-08 | B14 | B14-19 | AS0889 | 2016 | SA - Al-Baha | Grove | Mexican Lime |
|  | LR025-09 | B14 | B14-22 | AS0890 | 2016 | SA - Al-Baha | Grove | Mexican Lime |
|  | LR025-10 | B14 | B14-24 | AS0891 | 2016 | SA - Al-Baha | Grove | Mexican Lime |
|  | LR025-11 | B14 | B14-25 | AS0892 | 2016 | SA - Al-Baha | Grove | Mexican Lime |
|  | LR025-12 | B14 | B14-26 | AS0893 | 2016 | SA - Al-Baha | Grove | Mexican Lime |
|  | LR025-13 | B14 | B14-27 | AS0894 | 2016 | SA - Al-Baha | Grove | Mexican Lime |
|  | LR025-14 | B14 | B14-28 | AS0895 | 2016 | SA - Al-Baha | Grove | Mexican Lime |
|  | LR026-01 | B11 | B11-4 | AS0896 | 2016 | SA - Al-Baha | Grove | Mexican Lime |
|  | LR026-03 | B11 | B11-5 | AS0896 | 2016 | SA - Al-Baha | Grove | Mexican Lime |
|  | LR026-02 | B11 | B11-6 | AS0897 | 2016 | SA - Al-Baha | Grove | Mexican Lime |
|  | LR026-04 | B11 | B11-7 | AS0898 | 2016 | SA - Al-Baha | Grove | Mexican Lime |
|  | LR026-05 | B11 | B11-8 | AS0899 | 2016 | SA - Al-Baha | Grove | Mexican Lime |
|  | LR026-06 | B11 | B11-9 | AS0899 | 2016 | SA - Al-Baha | Grove | Mexican Lime |
|  | LR026-07 | B11 | B11-10 | AS0899 | 2016 | SA - Al-Baha | Grove | Mexican Lime |
|  | LR026-08 | B11 | B11-11 | AS0899 | 2016 | SA - Al-Baha | Grove | Mexican Lime |
|  | LR026-09 | B11 | B11-12 | AS0900 | 2016 | SA - Al-Baha | Grove | Mexican Lime |
|  | LR026-10 | B11 | B11-13 | AS0900 | 2016 | SA - Al-Baha | Grove | Mexican Lime |
|  | LR026-11 | B11 | B11-14 | AS0900 | 2016 | SA - Al-Baha | Grove | Mexican Lime |
|  | LR026-12 | B11 | B11-15 | AS0901 | 2016 | SA - Al-Baha | Grove | Mexican Lime |
|  | LR026-13 | B11 | B11-16 | AS0902 | 2016 | SA - Al-Baha | Grove | Mexican Lime |
|  | LR027-01 | JP4 | JP4-7 | AS0903 | 2014 | SA - Jizan | Grove | Mexican Lime |
|  | LR027-06 | JP4 | JP4-8 | AS0903 | 2014 | SA - Jizan | Grove | Mexican Lime |
|  | LR027-08 | JP4 | JP4-9 | AS0903 | 2014 | SA - Jizan | Grove | Mexican Lime |
|  | LR027-09 | JP4 | JP4-10 | AS0903 | 2014 | SA - Jizan | Grove | Mexican Lime |
|  | LR027-11 | JP4 | JP4-11 | AS0903 | 2014 | SA - Jizan | Grove | Mexican Lime |
|  | LR027-04 | JP4 | JP4-12 | AS0904 | 2014 | SA - Jizan | Grove | Mexican Lime |
|  | LR027-07 | JP4 | JP4-13 | AS0905 | 2014 | SA - Jizan | Grove | Mexican Lime |
|  | LR027-10 | JP4 | JP4-13 | AS0906 | 2014 | SA - Jizan | Grove | Mexican Lime |
|  | LR027-12 | JP4 | JP4-14 | AS0907 | 2014 | SA - Jizan | Grove | Mexican Lime |
|  | LR028-01 | B8 | B8-8 | AS0909 | 2016 | SA - Al-Baha | Grove | Mexican Lime |
|  | LR028-03 | B8 | B8-9 | AS0909 | 2016 | SA - Al-Baha | Grove | Mexican Lime |
|  | LR028-02 | B8 | B8-10 | AS0910 | 2016 | SA - Al-Baha | Grove | Mexican Lime |
|  | LR028-04 | B8 | B8-11 | AS0911 | 2016 | SA - Al-Baha | Grove | Mexican Lime |
|  | LR028-05 | B8 | B8-12 | AS0912 | 2016 | SA - Al-Baha | Grove | Mexican Lime |
|  | LR028-06 | B8 | B8-13 | AS0913 | 2016 | SA - Al-Baha | Grove | Mexican Lime |
|  | LR028-07 | B8 | B8-14 | AS0914 | 2016 | SA - Al-Baha | Grove | Mexican Lime |
|  | LR028-08 | B8 | B8-15 | AS0915 | 2016 | SA - Al-Baha | Grove | Mexican Lime |
|  | LR028-09 | B8 | B8-16 | AS0916 | 2016 | SA - Al-Baha | Grove | Mexican Lime |
|  | LR028-10 | B8 | B8-17 | AS0917 | 2016 | SA - Al-Baha | Grove | Mexican Lime |
|  | LR028-11 | B8 | B8-18 | AS0918 | 2016 | SA - Al-Baha | Grove | Mexican Lime |
|  | LR028-12 | B8 | B8-19 | AS0919 | 2016 | SA - Al-Baha | Grove | Mexican Lime |
|  | LR028-13 | B8 | B8-20 | AS0920 | 2016 | SA - Al-Baha | Grove | Mexican Lime |
|  | LR029-01 | YHP2 | YHP2-15 | AS0921 | 2014 | YE- Al-Hudaydah | Grove | Mexican Lime |
|  | LR029-02 | YHP2 | YHP2-16 | AS0922 | 2014 | YE- Al-Hudaydah | Grove | Mexican Lime |
|  | LR029-03 | YHP2 | YHP2-17 | AS0923 | 2014 | YE- Al-Hudaydah | Grove | Mexican Lime |
|  | LR029-05 | YHP2 | YHP2-18 | AS0924 | 2014 | YE- Al-Hudaydah | Grove | Mexican Lime |
|  | LR029-06 | YHP2 | YHP2-19 | AS0925 | 2014 | YE- Al-Hudaydah | Grove | Mexican Lime |
|  | LR029-07 | YHP2 | YHP2-20 | AS0926 | 2014 | YE- Al-Hudaydah | Grove | Mexican Lime |
|  | LR029-09 | YHP2 | YHP2-21 | AS0927 | 2014 | YE- Al-Hudaydah | Grove | Mexican Lime |
|  | LR029-10 | YHP2 | YHP2-22 | AS0928 | 2014 | YE- Al-Hudaydah | Grove | Mexican Lime |
|  | LR029-11 | YHP2 | YHP2-23 | AS0929 | 2014 | YE- Al-Hudaydah | Grove | Mexican Lime |
|  | LR029-12 | YHP2 | YHP2-24 | AS0930 | 2014 | YE- Al-Hudaydah | Grove | Mexican Lime |
|  | LR029-13 | YHP2 | YHP2-25 | AS0931 | 2014 | YE- Al-Hudaydah | Grove | Mexican Lime |
|  | LR029-14 | YHP2 | YHP2-26 | AS0932 | 2014 | YE- Al-Hudaydah | Grove | Mexican Lime |
|  | LR029-15 | YHP2 | YHP2-27 | AS0933 | 2014 | YE- Al-Hudaydah | Grove | Mexican Lime |
|  | LR029-16 | YHP2 | YHP2-28 | AS0934 | 2014 | YE- Al-Hudaydah | Grove | Mexican Lime |
|  | LR029-17 | YHP2 | YHP2-29 | AS0935 | 2014 | YE- Al-Hudaydah | Grove | Mexican Lime |
|  | LR029-18 | YHP2 | YHP2-30 | AS0936 | 2014 | YE- Al-Hudaydah | Grove | Mexican Lime |
|  | LR029-20 | YHP2 | YHP2-31 | AS0937 | 2014 | YE- Al-Hudaydah | Grove | Mexican Lime |
|  | LR030-01 | JP1 | JP1-5 | AS0938 | 2014 | SA – Jizan | Grove | Lemon |
|  | LR030-02 | JP1 | JP1-6 | AS0939 | 2014 | SA – Jizan | Grove | Lemon |
|  | LR030-03 | JP1 | JP1-7 | AS0940 | 2014 | SA – Jizan | Grove | Lemon |
|  | LR030-04 | JP1 | JP1-8 | AS0941 | 2014 | SA – Jizan | Grove | Lemon |
|  | LR030-05 | JP1 | JP1-9 | AS0942 | 2014 | SA – Jizan | Grove | Lemon |
|  | LR030-07 | JP1 | JP1-10 | AS0943 | 2014 | SA – Jizan | Grove | Lemon |
|  | LR030-08 | JP1 | JP1-11 | AS0944 | 2014 | SA – Jizan | Grove | Lemon |
|  | LR030-10***** | JP1 | JP1-12 | AS0945 | 2014 | SA – Jizan | Grove | Lemon |
|  | LR030-11 | JP1 | JP1-13 | AS0946 | 2014 | SA – Jizan | Grove | Lemon |
|  | LR030-12 | JP1 | JP1-14 | AS0947 | 2014 | SA – Jizan | Grove | Lemon |
|  | LR030-13 | JP1 | JP1-15 | AS0948 | 2014 | SA – Jizan | Grove | Lemon |
|  | LR030-14***** | JP1 | JP1-16 | AS0949 | 2014 | SA – Jizan | Grove | Lemon |
|  | LR030-15***** | JP1 | JP1-17 | AS0950 | 2014 | SA – Jizan | Grove | Lemon |
|  | LR030-16 | JP1 | JP1-18 | AS0951 | 2014 | SA – Jizan | Grove | Lemon |
|  | LR030-17***** | JP1 | JP1-19 | AS0952 | 2014 | SA – Jizan | Grove | Lemon |
|  | LR031-01***** | YLP3 | YLP3-4 | AS0953 | 2014 | YE- Lahj | Grove | Mexican Lime |
|  | LR031-02***** | YLP3 | YLP3-5 | AS0954 | 2014 | YE- Lahj | Grove | Mexican Lime |
|  | LR031-08 | YLP3 | YLP3-6 | AS0954 | 2014 | YE- Lahj | Grove | Mexican Lime |
|  | LR031-03 | YLP3 | YLP3-7 | AS0955 | 2014 | YE- Lahj | Grove | Mexican Lime |
|  | LR031-04 | YLP3 | YLP3-8 | AS0956 | 2014 | YE- Lahj | Grove | Mexican Lime |
|  | LR031-05***** | YLP3 | YLP3-9 | AS0957 | 2014 | YE- Lahj | Grove | Mexican Lime |
|  | LR031-06 | YLP3 | YLP3-10 | AS0958 | 2014 | YE- Lahj | Grove | Mexican Lime |
|  | LR031-07 | YLP3 | YLP3-11 | AS0959 | 2014 | YE- Lahj | Grove | Mexican Lime |
|  | LR031-09 | YLP3 | YLP3-12 | AS0960 | 2014 | YE- Lahj | Grove | Mexican Lime |
|  | LR031-10 | YLP3 | YLP3-13 | AS0961 | 2014 | YE- Lahj | Grove | Mexican Lime |
|  | LR031-11 | YLP3 | YLP3-14 | AS0962 | 2014 | YE- Lahj | Grove | Mexican Lime |
|  | LR032-01 | YHP1 | YHP1-8 | AS0963 | 2014 | YE- Al-Hudaydah | Grove | Mexican Lime |
|  | LR032-02 | YHP1 | YHP1-9 | AS0964 | 2014 | YE- Al-Hudaydah | Grove | Mexican Lime |
|  | LR032-06 | YHP1 | YHP1-10 | AS0964 | 2014 | YE- Al-Hudaydah | Grove | Mexican Lime |
|  | LR032-03 | YHP1 | YHP1-11 | AS0965 | 2014 | YE- Al-Hudaydah | Grove | Mexican Lime |
|  | LR032-04 | YHP1 | YHP1-12 | AS0966 | 2014 | YE- Al-Hudaydah | Grove | Mexican Lime |
|  | LR032-05 | YHP1 | YHP1-13 | AS0967 | 2014 | YE- Al-Hudaydah | Grove | Mexican Lime |
|  | LR032-07 | YHP1 | YHP1-14 | AS0968 | 2014 | YE- Al-Hudaydah | Grove | Mexican Lime |
|  | LR032-08 | YHP1 | YHP1-15 | AS0969 | 2014 | YE- Al-Hudaydah | Grove | Mexican Lime |
|  | LR032-09 | YHP1 | YHP1-16 | AS0970 | 2014 | YE- Al-Hudaydah | Grove | Mexican Lime |
|  | LR032-11 | YHP1 | YHP1-17 | AS0971 | 2014 | YE- Al-Hudaydah | Grove | Mexican Lime |
|  | LR032-12 | YHP1 | YHP1-18 | AS0972 | 2014 | YE- Al-Hudaydah | Grove | Mexican Lime |
|  | LR032-13 | YHP1 | YHP1-19 | AS0973 | 2014 | YE- Al-Hudaydah | Grove | Mexican Lime |
|  | LR032-14 | YHP1 | YHP1-20 | AS0974 | 2014 | YE- Al-Hudaydah | Grove | Mexican Lime |
|  | LR032-16 | YHP1 | YHP1-21 | AS0975 | 2014 | YE- Al-Hudaydah | Grove | Mexican Lime |
|  | LR032-17 | YHP1 | YHP1-22 | AS0976 | 2014 | YE- Al-Hudaydah | Grove | Mexican Lime |
|  | LR032-18 | YHP1 | YHP1-23 | AS0977 | 2014 | YE- Al-Hudaydah | Grove | Mexican Lime |
|  | LR032-19 | YHP1 | YHP1-24 | AS0978 | 2014 | YE- Al-Hudaydah | Grove | Mexican Lime |
|  | LR032-20 | YHP1 | YHP1-25 | AS0979 | 2014 | YE- Al-Hudaydah | Grove | Mexican Lime |
|  | LR032-21 | YHP1 | YHP1-26 | AS0980 | 2014 | YE- Al-Hudaydah | Grove | Mexican Lime |
|  | LR032-22 | YHP1 | YHP1-27 | AS0980 | 2014 | YE- Al-Hudaydah | Grove | Mexican Lime |
|  | LR032-23 | YHP1 | YHP1-28 | AS0981 | 2014 | YE- Al-Hudaydah | Grove | Mexican Lime |
|  | LR032-24 | YHP1 | YHP1-29 | AS0982 | 2014 | YE- Al-Hudaydah | Grove | Mexican Lime |
|  | LR033-01 | YHP3 | YHP3-4 | AS0983 | 2014 | YE- Al-Hudaydah | Grove | Mexican Lime |
|  | LR033-02 | YHP3 | YHP3-5 | AS0984 | 2014 | YE- Al-Hudaydah | Grove | Mexican Lime |
|  | LR033-03 | YHP3 | YHP3-6 | AS0985 | 2014 | YE- Al-Hudaydah | Grove | Mexican Lime |
|  | LR033-04 | YHP3 | YHP3-7 | AS0986 | 2014 | YE- Al-Hudaydah | Grove | Mexican Lime |
|  | LR033-05 | YHP3 | YHP3-8 | AS0987 | 2014 | YE- Al-Hudaydah | Grove | Mexican Lime |
|  | LR033-08 | YHP3 | YHP3-9 | AS0988 | 2014 | YE- Al-Hudaydah | Grove | Mexican Lime |
|  | LR033-09 | YHP3 | YHP3-10 | AS0989 | 2014 | YE- Al-Hudaydah | Grove | Mexican Lime |
|  | LR033-10 | YHP3 | YHP3-11 | AS0990 | 2014 | YE- Al-Hudaydah | Grove | Mexican Lime |
|  | LR033-12 | YHP3 | YHP3-12 | AS0991 | 2014 | YE- Al-Hudaydah | Grove | Mexican Lime |
|  | LR033-13 | YHP3 | YHP3-13 | AS0992 | 2014 | YE- Al-Hudaydah | Grove | Mexican Lime |
|  | LR033-14 | YHP3 | YHP3-14 | AS0993 | 2014 | YE- Al-Hudaydah | Grove | Mexican Lime |
|  | LR033-15 | YHP3 | YHP3-15 | AS0994 | 2014 | YE- Al-Hudaydah | Grove | Mexican Lime |
|  | LR033-16 | YHP3 | YHP3-16 | AS0995 | 2014 | YE- Al-Hudaydah | Grove | Mexican Lime |
|  | LR033-17 | YHP3 | YHP3-17 | AS0996 | 2014 | YE- Al-Hudaydah | Grove | Mexican Lime |
|  | LR033-18 | YHP3 | YHP3-18 | AS0997 | 2014 | YE- Al-Hudaydah | Grove | Mexican Lime |
|  | LR034-01 | JP2 | JP2-4 | AS0998 | 2014 | SA – Jizan | Grove | Mexican Lime |
|  | LR034-02 | JP2 | JP2-5 | AS0999 | 2014 | SA – Jizan | Grove | Mexican Lime |
|  | LR034-04 | JP2 | JP2-6 | AS1000 | 2014 | SA – Jizan | Grove | Mexican Lime |
|  | LR034-05 | JP2 | JP2-7 | AS1001 | 2014 | SA – Jizan | Grove | Mexican Lime |
|  | LR034-06 | JP2 | JP2-8 | AS1002 | 2014 | SA – Jizan | Grove | Mexican Lime |
|  | LR034-07 | JP2 | JP2-9 | AS1003 | 2014 | SA – Jizan | Grove | Mexican Lime |
|  | LR034-08 | JP2 | JP2-10 | AS1004 | 2014 | SA – Jizan | Grove | Mexican Lime |
|  | LR034-09 | JP2 | JP2-11 | AS1005 | 2014 | SA – Jizan | Grove | Mexican Lime |
|  | LR034-10 | JP2 | JP2-13 | AS1006 | 2014 | SA – Jizan | Grove | Mexican Lime |
|  | LR034-11 | JP2 | JP2-14 | AS1007 | 2014 | SA – Jizan | Grove | Mexican Lime |
|  | LR034-12 | JP2 | JP2-16 | AS1008 | 2014 | SA - Jizan | Grove | Mexican Lime |
|  | LR034-13 | JP2 | JP2-17 | AS1009 | 2014 | SA - Jizan | Grove | Mexican Lime |
|  | LR034-14 | JP2 | JP2-18 | AS1010 | 2014 | SA - Jizan | Grove | Mexican Lime |
|  | LR034-15 | JP2 | JP2-19 | AS1011 | 2014 | SA - Jizan | Grove | Mexican Lime |
|  | LR035-01***** | YTP1 | YTP1-5 | AS1012 | 2014 | YE- Taiz | Grove | Mexican Lime |
|  | LR035-02***** | YTP1 | YTP1-6 | AS1013 | 2014 | YE- Taiz | Grove | Mexican Lime |
|  | LR035-03 | YTP1 | YTP1-7 | AS1014 | 2014 | YE- Taiz | Grove | Mexican Lime |
|  | LR035-04 | YTP1 | YTP1-8 | AS1015 | 2014 | YE- Taiz | Grove | Mexican Lime |
|  | LR035-05 | YTP1 | YTP1-9 | AS1016 | 2014 | YE- Taiz | Grove | Mexican Lime |
|  | LR035-07 | YTP1 | YTP1-10 | AS1017 | 2014 | YE- Taiz | Grove | Mexican Lime |
|  | LR035-08***** | YTP1 | YTP1-11 | AS1018 | 2014 | YE- Taiz | Grove | Mexican Lime |
|  | LR033-11 | YHP3 | YHP3-19 | AS1019 | 2014 | YE- Al-Hudaydah | Grove | Mexican Lime |
|  | LR036-01 | YLP2 | YLP2-5 | AS1020 | 2014 | YE- Lahj | Grove | Mexican Lime |
|  | LR036-02 | YLP2 | YLP2-6 | AS1021 | 2014 | YE- Lahj | Grove | Mexican Lime |
|  | LR036-03 | YLP2 | YLP2-7 | AS1022 | 2014 | YE- Lahj | Grove | Mexican Lime |
|  | LR036-04 | YLP2 | YLP2-8 | AS1023 | 2014 | YE- Lahj | Grove | Mexican Lime |
|  | LR036-05 | YLP2 | YLP2-9 | AS1024 | 2014 | YE- Lahj | Grove | Mexican Lime |
|  | LR036-06 | YLP2 | YLP2-10 | AS1025 | 2014 | YE- Lahj | Grove | Mexican Lime |
|  | LR036-07 | YLP2 | YLP2-11 | AS1026 | 2014 | YE- Lahj | Grove | Mexican Lime |
|  | LR036-09 | YLP2 | YLP2-12 | AS1027 | 2014 | YE- Lahj | Grove | Mexican Lime |
|  | LR036-10 | YLP2 | YLP2-13 | AS1028 | 2014 | YE- Lahj | Grove | Mexican Lime |
|  | LR036-11 | YLP2 | YLP2-14 | AS1029 | 2014 | YE- Lahj | Grove | Mexican Lime |
|  | LR037-01 | YTP2 | YTP2-7 | AS1030 | 2014 | YE- Taiz | Grove | Mexican Lime |
|  | LR037-02 | YTP2 | YTP2-8 | AS1031 | 2014 | YE- Taiz | Grove | Mexican Lime |
|  | LR037-03 | YTP2 | YTP2-9 | AS1032 | 2014 | YE- Taiz | Grove | Mexican Lime |
|  | LR037-04***** | YTP2 | YTP2-10 | AS1033 | 2014 | YE- Taiz | Grove | Mexican Lime |
|  | LR037-05 | YTP2 | YTP2-11 | AS1034 | 2014 | YE- Taiz | Grove | Mexican Lime |
|  | LR037-06 | YTP2 | YTP2-12 | AS1035 | 2014 | YE- Taiz | Grove | Mexican Lime |
|  | LR037-07 | YTP2 | YTP2-13 | AS1036 | 2014 | YE- Taiz | Grove | Mexican Lime |
|  | LR038-02 | JP8 | JP8-7 | AS1037 | 2014 | SA – Jizan | Grove | Mexican Lime |
|  | LR038-03 | JP8 | JP8-8 | AS1038 | 2014 | SA – Jizan | Grove | Mexican Lime |
|  | LR038-04 | JP8 | JP8-9 | AS1039 | 2014 | SA – Jizan | Grove | Mexican Lime |
|  | LR038-05***** | JP8 | JP8-10 | AS1040 | 2014 | SA – Jizan | Grove | Mexican Lime |
|  | LR038-06 | JP8 | JP8-12 | AS1041 | 2014 | SA – Jizan | Grove | Mexican Lime |
|  | LR038-07 | JP8 | JP8-13 | AS1042 | 2014 | SA – Jizan | Grove | Mexican Lime |
|  | LR038-08 | JP8 | JP8-15 | AS1043 | 2014 | SA – Jizan | Grove | Mexican Lime |
|  | LR038-09 | JP8 | JP8-16 | AS1044 | 2014 | SA – Jizan | Grove | Mexican Lime |
|  | LR038-10 | JP8 | JP8-17 | AS1045 | 2014 | SA – Jizan | Grove | Mexican Lime |
|  | LR039-01 | JP9 | JP9-5 | AS1046 | 2014 | SA – Jizan | Grove | Mexican Lime |
|  | LR039-02 | JP9 | JP9-6 | AS1047 | 2014 | SA – Jizan | Grove | Mexican Lime |
|  | LR039-03 | JP9 | JP9-7 | AS1048 | 2014 | SA – Jizan | Grove | Mexican Lime |
|  | LR039-04 | JP9 | JP9-8 | AS1048 | 2014 | SA – Jizan | Grove | Mexican Lime |
|  | LR039-05 | JP9 | JP9-9 | AS1049 | 2014 | SA – Jizan | Grove | Mexican Lime |
|  | LR039-06 | JP9 | JP9-10 | AS1050 | 2014 | SA – Jizan | Grove | Mexican Lime |
|  | LR039-07 | JP9 | JP9-11 | AS1051 | 2014 | SA – Jizan | Grove | Mexican Lime |
|  | LR039-08 | JP9 | JP9-12 | AS1052 | 2014 | SA – Jizan | Grove | Mexican Lime |
|  | LR039-09 | JP9 | JP9-13 | AS1053 | 2014 | SA – Jizan | Grove | Mexican Lime |
|  | LR039-10 | JP9 | JP9-14 | AS1054 | 2014 | SA – Jizan | Grove | Mexican Lime |
|  | LR039-11 | JP9 | JP9-15 | AS1055 | 2014 | SA – Jizan | Grove | Mexican Lime |
|  | LR039-12 | JP9 | JP9-16 | AS1056 | 2014 | SA – Jizan | Grove | Mexican Lime |
|  | LR039-13 | JP9 | JP9-17 | AS1057 | 2014 | SA – Jizan | Grove | Mexican Lime |
|  | LR039-14 | JP9 | JP9-18 | AS1057 | 2014 | SA – Jizan | Grove | Mexican Lime |
|  | LR039-15 | JP9 | JP9-19 | AS1058 | 2014 | SA – Jizan | Grove | Mexican Lime |
|  | LR040-01 | YLP1 | YLP1-6 | AS1059 | 2014 | YE- Lahj | Grove | Mexican Lime |
|  | LR040-02 | YLP1 | YLP1-7 | AS1060 | 2014 | YE- Lahj | Grove | Mexican Lime |
|  | LR040-03 | YLP1 | YLP1-8 | AS1060 | 2014 | YE- Lahj | Grove | Mexican Lime |
|  | LR040-05 | YLP1 | YLP1-9 | AS1061 | 2014 | YE- Lahj | Grove | Mexican Lime |
|  | LR041-01 | YLP4 | YLP4-9 | AS1062 | 2014 | YE- Lahj | Grove | Mexican Lime |
|  | LR041-02 | YLP4 | YLP4-10 | AS1063 | 2014 | YE- Lahj | Grove | Mexican Lime |
|  | LR042-01 | JP6 | JP6-4 | AS1064 | 2014 | SA – Jizan | Grove | Mexican Lime |
|  | LR042-02 | JP6 | JP6-5 | AS1065 | 2014 | SA – Jizan | Grove | Mexican Lime |
|  | LR042-12 | JP6 | JP6-6 | AS1065 | 2014 | SA – Jizan | Grove | Mexican Lime |
|  | LR044-05 | JP7 | JP7-7 | AS1065 | 2014 | SA – Jizan | Grove | Mexican Lime |
|  | LR044-06 | JP7 | JP7-8 | AS1065 | 2014 | SA – Jizan | Grove | Mexican Lime |
|  | LR042-03 | JP6 | JP6-7 | AS1066 | 2014 | SA – Jizan | Grove | Mexican Lime |
|  | LR042-04 | JP6 | JP6-8 | AS1067 | 2014 | SA – Jizan | Grove | Mexican Lime |
|  | LR042-05 | JP6 | JP6-9 | AS1068 | 2014 | SA – Jizan | Grove | Mexican Lime |
|  | LR042-06 | JP6 | JP6-10 | AS1069 | 2014 | SA – Jizan | Grove | Mexican Lime |
|  | LR042-07 | JP6 | JP6-12 | AS1070 | 2014 | SA – Jizan | Grove | Mexican Lime |
|  | LR042-10 | JP6 | JP6-13 | AS1070 | 2014 | SA – Jizan | Grove | Mexican Lime |
|  | LR042-08 | JP6 | JP6-15 | AS1071 | 2014 | SA – Jizan | Grove | Mexican Lime |
|  | LR042-09 | JP6 | JP6-16 | AS1072 | 2014 | SA – Jizan | Grove | Mexican Lime |
|  | LR011-20 | B1 | B1-22 | AS1073 | 2016 | SA - Al-Baha | Grove | Mexican Lime |
|  | LR042-11 | JP6 | JP6-17 | AS1074 | 2014 | SA – Jizan | Grove | Mexican Lime |
|  | LR042-13 | JP6 | JP6-18 | AS1075 | 2014 | SA – Jizan | Grove | Mexican Lime |
|  | LR042-14 | JP6 | JP6-19 | AS1076 | 2014 | SA – Jizan | Grove | Mexican Lime |
|  | LR043-01 | ABP2 | ABP2-12 | AS1077 | 2015 | SA – Aseer | Grove | Sweet Orange |
|  | LR043-02***** | ABP2 | ABP2-13 | AS1078 | 2015 | SA – Aseer | Grove | Sweet Orange |
|  | LR044-01 | JP7 | JP7-9 | AS1079 | 2014 | SA – Jizan | Grove | Mexican Lime |
|  | LR044-02 | JP7 | JP7-10 | AS1080 | 2014 | SA – Jizan | Grove | Mexican Lime |
|  | LR044-03 | JP7 | JP7-11 | AS1081 | 2014 | SA – Jizan | Grove | Mexican Lime |
|  | LR044-08 | JP7 | JP7-12 | AS1081 | 2014 | SA – Jizan | Grove | Mexican Lime |
|  | LR044-09 | JP7 | JP7-13 | AS1081 | 2014 | SA – Jizan | Grove | Mexican Lime |
|  | LR044-04 | JP7 | JP7-14 | AS1082 | 2014 | SA – Jizan | Grove | Mexican Lime |
|  | LR044-07 | JP7 | JP7-15 | AS1083 | 2014 | SA – Jizan | Grove | Mexican Lime |
|  | LR044-10 | JP7 | JP7-16 | AS1084 | 2014 | SA – Jizan | Grove | Mexican Lime |
|  | LR044-11 | JP7 | JP7-17 | AS1085 | 2014 | SA – Jizan | Grove | Mexican Lime |
|  | LR045-01 | JP10 | JP10-3 | AS1086 | 2014 | SA – Jizan | Grove | Mexican Lime |
|  | LR045-02***** | JP10 | JP10-4 | AS1087 | 2014 | SA – Jizan | Grove | Mexican Lime |
|  | LR045-03 | JP10 | JP10-5 | AS1088 | 2014 | SA – Jizan | Grove | Mexican Lime |
|  | LR045-04 | JP10 | JP10-6 | AS1089 | 2014 | SA – Jizan | Grove | Mexican Lime |
|  | LR045-05 | JP10 | JP10-7 | AS1090 | 2014 | SA – Jizan | Grove | Mexican Lime |
|  | LR045-06 | JP10 | JP10-8 | AS1091 | 2014 | SA – Jizan | Grove | Mexican Lime |
|  | LR045-07 | JP10 | JP10-9 | AS1092 | 2014 | SA – Jizan | Grove | Mexican Lime |
|  | LR045-08 | JP10 | JP10-10 | AS1093 | 2014 | SA – Jizan | Grove | Mexican Lime |
|  | LR045-09 | JP10 | JP10-11 | AS1094 | 2014 | SA – Jizan | Grove | Mexican Lime |
|  | LR045-10***** | JP10 | JP10-12 | AS1095 | 2014 | SA – Jizan | Grove | Mexican Lime |
|  | LR046-02 | B12 | B12-5 | AS1096 | 2016 | SA - Al-Baha | Grove | Mexican Lime |
|  | LR046-03 | B12 | B12-6 | AS1097 | 2016 | SA - Al-Baha | Grove | Mexican Lime |
|  | LR047-01 | YT1 | YT1 | AS1098 | 2014 | YE- Taiz | Grove | Mexican Lime |
|  | LR047-02 | YT2 | YT2 | AS1099 | 2014 | YE- Taiz | Grove | Mexican Lime |
|  | LR048-01 | YLP5 | YLP5-5 | AS1100 | 2014 | YE- Lahj | Grove | Mexican Lime |
|  | LR011-12 | B1 | B1-23 | AS1102 | 2016 | SA - Al-Baha | Grove | Mexican Lime |
|  | LR012-15B | B4 | B4-24 | AS1103 | 2016 | SA - Al-Baha | Grove | Mexican Lime |
|  | LR012-16 | B4 | B4-25 | AS1104 | 2016 | SA - Al-Baha | Grove | Mexican Lime |
|  | LR012-18 | B4 | B4-26 | AS1105 | 2016 | SA - Al-Baha | Grove | Mexican Lime |
|  | LR018-10B | B9 | B9-28 | AS1105 | 2016 | SA - Al-Baha | Grove | Mexican Lime |
|  | LR016-19 | B2 | B2-26 | AS1106 | 2016 | SA - Al-Baha | Grove | Mexican Lime |
|  | LR016-20 | B2 | B2-27 | AS1107 | 2016 | SA - Al-Baha | Grove | Mexican Lime |
|  | LR017-31 | B6 | B6-33 | AS1108 | 2016 | SA - Al-Baha | Grove | Mexican Lime |
|  | LR017-32 | B6 | B6-34 | AS1109 | 2016 | SA - Al-Baha | Grove | Mexican Lime |
|  | LR018-19 | B9 | B9-29 | AS1110 | 2016 | SA - Al-Baha | Grove | Mexican Lime |
|  | LR018-23 | B9 | B9-30 | AS1111 | 2016 | SA - Al-Baha | Grove | Mexican Lime |
|  | LR018-26 | B9 | B9-31 | AS1112 | 2016 | SA - Al-Baha | Grove | Mexican Lime |
|  | LR021-07 | B3 | B3-8 | AS1113 | 2016 | SA - Al-Baha | Grove | Mexican Lime |
|  | LR021-08 | B3 | B3-9 | AS1114 | 2016 | SA - Al-Baha | Grove | Mexican Lime |
|  | LR023-17 | JP5 | JP5-21 | AS1115 | 2014 | SA – Jizan | Grove | Mexican Lime |
|  | LR025-04 | B14 | B14-29 | AS1116 | 2016 | SA - Al-Baha | Grove | Mexican Lime |
|  | LR028-14 | B8 | B8-21 | AS1117 | 2016 | SA - Al-Baha | Grove | Mexican Lime |
|  | LR028-15 | B8 | B8-22 | AS1118 | 2016 | SA - Al-Baha | Grove | Mexican Lime |
|  | LR032-10 | YHP1 | YHP1-30 | AS1119 | 2014 | YE- Al-Hudaydah | Grove | Mexican Lime |
|  | LR036-08 | YLP2 | YLP2-15 | AS1120 | 2014 | YE- Lahj | Grove | Mexican Lime |
|  | LR039-17 | JP9 | JP9-20 | AS1121 | 2014 | SA – Jizan | Grove | Mexican Lime |
|  | LR040-04 | YLP1 | YLP1-10 | AS1122 | 2014 | YE- Lahj | Grove | Mexican Lime |
|  | LR012-19 | B4 | B4-27 | AS1123 | 2016 | SA - Al-Baha | Grove | Mexican Lime |
|  | LR034-03 | JP2 | JP2-20 | AS1124 | 2014 | SA – Jizan | Grove | Mexican Lime |
|  | LR016-02 | B2 | B2-28 | AS1125 | 2016 | SA - Al-Baha | Grove | Mexican Lime |
|  | LR017-17 | B6 | B6-35 | AS1126 | 2016 | SA - Al-Baha | Grove | Mexican Lime |
|  | LR018-18 | B9 | B9-32 | AS1127 | 2016 | SA - Al-Baha | Grove | Mexican Lime |
|  | LR018-25 | B9 | B9-33 | AS1128 | 2016 | SA - Al-Baha | Grove | Mexican Lime |
|  | LR025-17 | B14 | B14-30 | AS1129 | 2016 | SA - Al-Baha | Grove | Mexican Lime |
|  | LR027-05 | JP4 | JP4-15 | AS1130 | 2014 | SA – Jizan | Grove | Mexican Lime |
|  | LR029-08 | YHP2 | YHP2-32 | AS1131 | 2014 | YE- Al-Hudaydah | Grove | Mexican Lime |
|  | LR038-01 | JP8 | JP8-18 | AS1132 | 2014 | SA – Jizan | Grove | Mexican Lime |
|  | JJ185 | NA | JJ185 | AS1133 | 1982 | YE | Grove | NA |
|  | JK004-08 | NA | JK004-08 | AS1134 | 1988 | YE | Grove | NA |
|  | JK004-09 | NA | JK004-09 | AS1135 | 1988 | YE | Grove | NA |

^1^Saudi Arabia

^2^Yemen

^3^not available

*Strains were tested for copper resistance and pathogenicity on Mexican lime and mandarin cv.Kinnow
