# Supplementary material for: Molecular epidemiology of the citrus bacterial pathogen Xanthomonas citri pv. citri from the Arabian Peninsula reveals a complex structure of specialist and generalist strains
Source: Evol Appl. 2022 Aug 26;15(9):1423–35. doi: 10.1111/eva.13451 (PMC9488683; doi:10.1111/eva.13451)
Supplement: Supplementary file 4 — Appendix S1 [file EVA-15-1423-s001.docx]

**Fig. S1:** Minimum spanning tree from MLVA‐31 data showing the genetic diversity of *X. citri* pv. *citri* pathotype A*. All strains from distinct networks or singletons differed at ≥ 3 minisatellite loci. Dots represent haplotypes. Dot diameter and color are representative of the number of strains per haplotype, and country of isolation, respectively (blue: Iran; red: Saudi Arabia; green: Oman; lavender: Yemen; pink: Thailand; violet: Cambodia; orange: India; grey: Pakistan; Yellow: Mauritius; salmon pink: Réunion; cyan: Comoros; black: Fiji).

**Fig. S2**: Genetic structure of *Xanthomonas citri* pv. *citri* lineage 4 (pathotype A*) in Saudi Arabia and Yemen based on the discriminant analysis of principal components (DAPC) of microsatellite data. Numbers and colors represent the nine genetic clusters retained from Bayesian information criterion (BIC) values. (A) Scatterplot representing axes 1 and 2 of the DAPC. (B) Scatterplot representing axes 1 and 3 of the DAPC. (C) Scatterplot representing axes 1 and 4 of the DAPC.
